# Comparative Structural Behaviour of Periodic Two-Layer Spatial Grids Inspired by Ammann A4 Geometry

**DOI:** 10.3390/ma19214461

**Published:** 2026-10-23

**Authors:** Romuald Tarczewski, Marcin Brzezicki

**Affiliations:** Faculty of Architecture, Wroclaw University of Science and Technology, Prusa 53/55, 50-317 Wrocław, Poland; marcin.brzezicki@pwr.edu.pl

**Keywords:** Ammann A4 aperiodic tessellation, double-layer spatial grids, geometric rearrangement of prototiles, Ammann-line-based lower subsystem, comparative structural analysis

## Abstract

This study investigates the structural behaviour of three periodic two-layer spatial grids inspired by Ammann A4 geometry. The proposed configurations combine tile-derived upper grids with lower subsystems formed by selected continuous Ammann lines, interconnected through diagonal members. These lines, which originally served as an auxiliary mathematical construction ensuring aperiodicity, are utilized here as the generator for a completely new concept of the grid’s bottom layer. Variants V1–V3 were compared with Ammann A4 and Unibat reference configurations using geometrically nonlinear finite element analysis under common loading, material, and member-section assumptions. Axial forces, resultant bending moments, and vertical displacements were evaluated across the configurations and their constituent subsystems. All lower-subsystem members in V1–V3 carried tensile axial forces. V1 exhibited the most uniform lower-subsystem force distribution, with a coefficient of variation of 4.0%, and the lowest peak axial-force magnitude and resultant bending moment among the proposed variants, at 324.68 kN and 72.57 kNm, respectively. V2 developed the highest corresponding peaks, at 448.32 kN and 129.12 kNm. V3 achieved the smallest maximum downward displacement among the proposed variants, at 18.60 cm, compared with 30.70 cm for V1 and 24.70 cm for V2. Unibat exhibited substantially smaller bending moments and displacements but required greater total member length and used a different support arrangement. The findings demonstrate that Ammann-line-based lower subsystems can develop continuous tensile paths, while revealing trade-offs between force uniformity, local demand, and deformation. The comparison characterises relative structural response rather than establishing code compliance or a universally preferable configuration.

## 1. Introduction

Tilings are usually discussed as two-dimensional systems of geometric order, but their internal logic can also be seen as a generator of spatial structural configurations. The boundaries, adjacencies, and directional continuities of tiles define not only a surface pattern but also a potential graph of nodes and members through which force paths may develop. From this perspective, a tiling may serve as a starting point for developing structural configurations in which geometry, topology, and load transfer are considered together. This interpretation is particularly relevant for spatial systems in which the distribution of members, the continuity of tensile paths, and the coupling between layers are determined by an underlying geometric rule rather than by a conventional orthogonal grid [1,2,3].

Aperiodic tile sets provide a particularly relevant reference point for such an interpretation. Among the best-known planar examples are the tilings developed by Roger Penrose in the 1970s and independently, shortly thereafter by Robert Ammann [2]. Unlike periodic tilings, an aperiodic tiling admits no non-zero translational symmetry, although particular configurations may exhibit non-crystallographic rotational symmetries, including fivefold symmetry. Penrose developed three principal families of aperiodic tilings. The earliest, commonly designated as P1, uses a larger set of prototiles, whereas the later P2 and P3 systems can each be generated from only two prototiles: kites and darts in P2, and thick and thin rhombs in P3.

During the 1970s, Robert Ammann developed several additional sets of aperiodic prototiles, subsequently described and systematised in the literature [2,4]. These sets are conventionally designated by the letter A followed by a numeral, as in the A4 set considered in this study. An important property of Ammann tilings is their hierarchical organisation, expressed through inflation and deflation: admissible groups of prototiles at one scale can be combined to form larger, geometrically related supertiles at the next hierarchical level [2,4,5]. This relationship between local matching conditions and larger-scale geometric order is particularly relevant to the structural interpretation explored in the present study.

**Figure 1:**
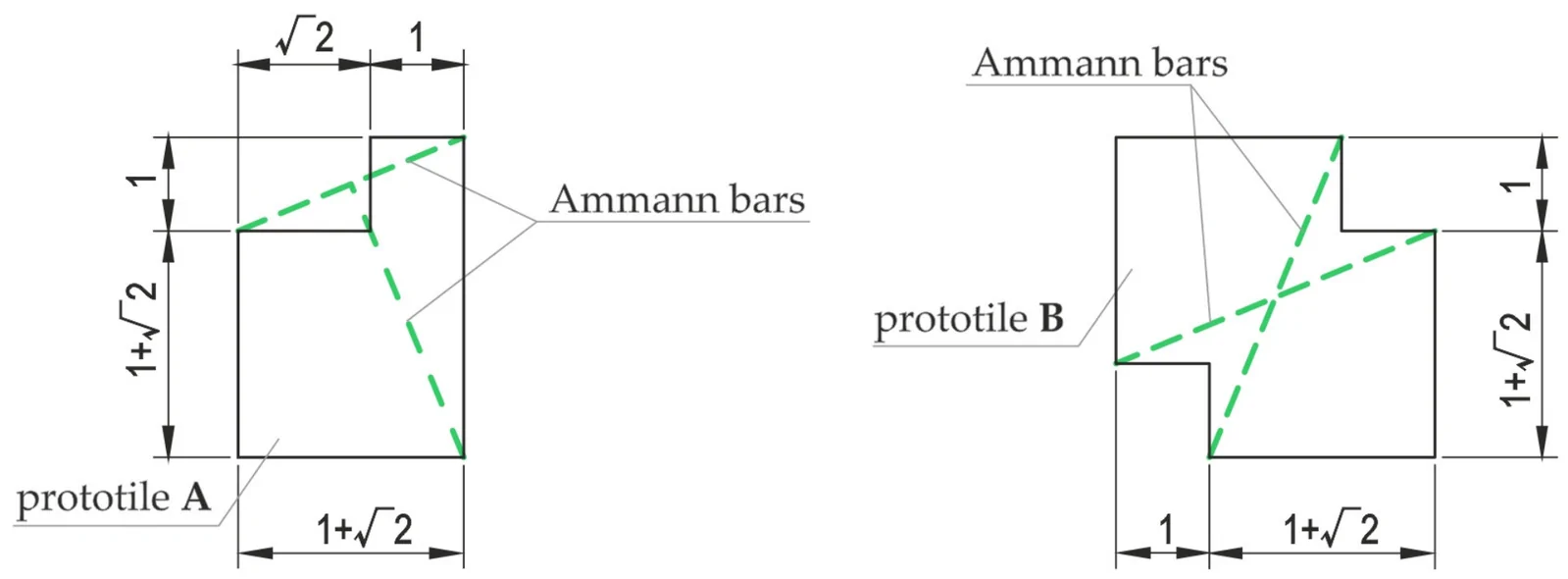
Basic prototiles forming Ammann A4 set flooring with the Ammann bars assigned to them marked in green: (**left**) tile A; (**right**) tile B.

The Ammann A4 set, described by Ammann, Grünbaum, and Shephard [2], consists of two prototiles, conventionally designated A and B, whose admissible arrangements are constrained by matching markings known as Ammann bars, as shown in Figure 1.

**Figure 2:**
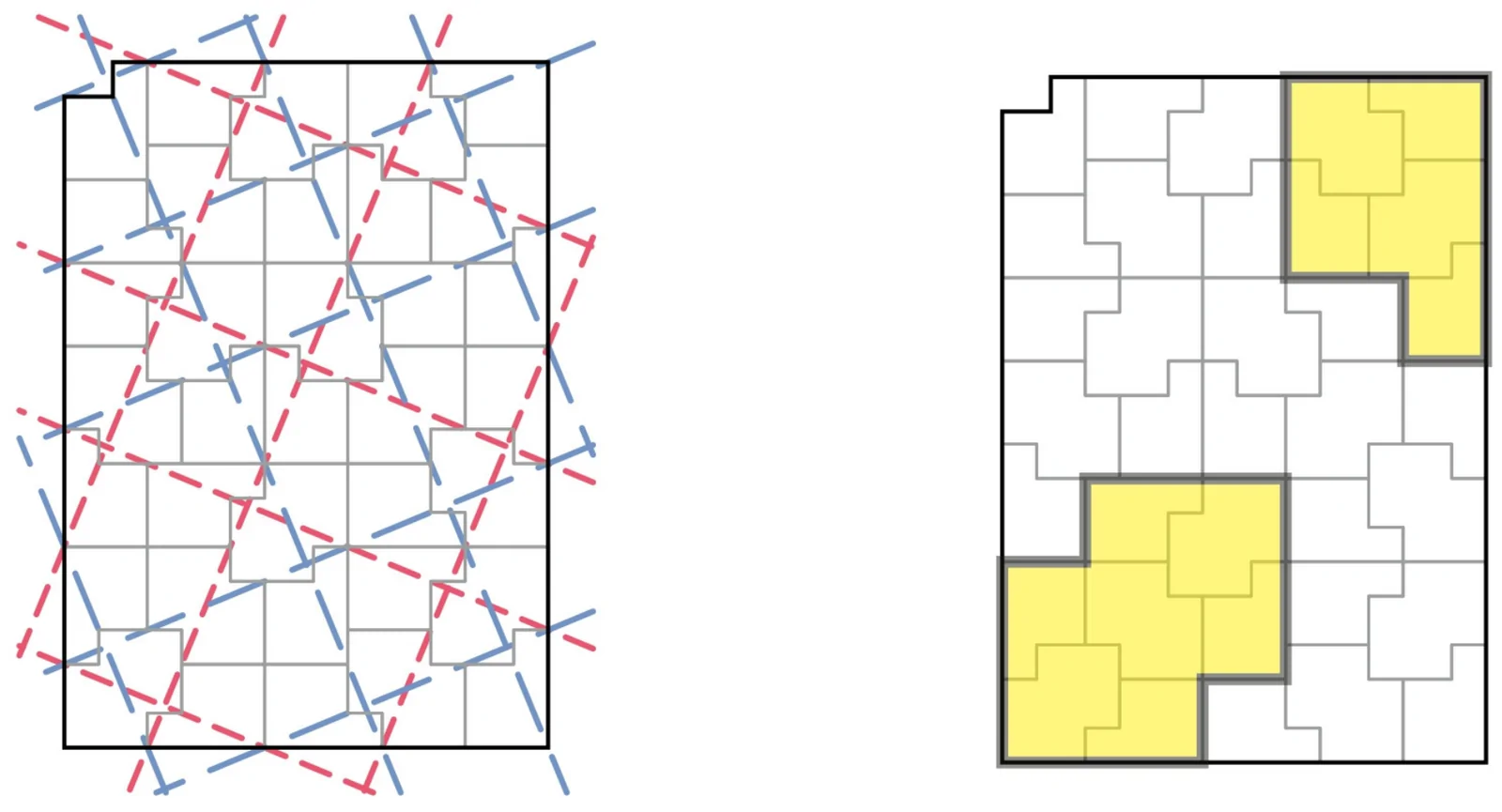
A fragment of the complete Ammann A4 tiling: (**left**) the A4 set with Ammann lines (in red and blue to show two families of those lines) formed by Ammann bars assigned to the individual prototiles; (**right**) hierarchical organisation of the Ammann tiling: the larger units highlighted in yellow are composed of smaller Ammann tiles, illustrating the emergence of the next hierarchical level within the pattern.

In the original mathematical formulation, these markings (Ammann bars) are not merely graphic features but form part of the local matching conditions that enforce aperiodic order. These bars are segments located within the repeating elements and are marked with red and blue lines in Figure 1. The rule for arranging tiles with Ammann bars is that they must form straight lines across the entire pattern, as shown in Figure 2 (left). Adhering to this condition ensures the aperiodicity of the resulting pattern [4]. The lines formed by connecting Ammann bars are known as Ammann lines, and they form two families of orthogonal grids, marked in red and blue in Figure 2 (left).

Compatible arrangements of the prototiles can be recursively assembled into larger A- and B-supertiles, preserving the matching relationships at successive hierarchical levels, Figure 2 (right). The A4 set should therefore be understood as a system in which local geometric constraints generate long-range aperiodic organisation through recursive hierarchical structure [2,6,7,8,9].

Beyond their mathematical formulation, aperiodic tilings have also been explored as generators of architectural and structural configurations (e.g., roofs). Shea [1] investigated an aperiodic spatial tiling as a design generator, while Tarczewski [5] considered Ammann bars in relation to aperiodic structural lattices and plate–lattice dualism. More directly at the scale of spatial lattice structures, Durn and Behnejad applied the Penrose kite-and-dart tiling to spherical lattice caps and examined their feasibility in terms of component regularity in comparison with conventional periodic configurations [10]. Recent studies have further demonstrated the mechanical relevance of aperiodic order in lattice and cellular systems. Moat et al. [11] investigated the compressive behaviour of cellular structures derived from Penrose P3 tilings, while Imediegwu et al. [12] examined the mechanical characteristics of a broader family of aperiodic lattices. More recently, Qi et al. investigated aperiodic but ordered cellular materials inspired by three types of Penrose tilings using finite element simulations and quasi-static compression experiments, demonstrating that differences in topological configuration lead to distinct deformation and failure mechanisms [13]. Together, these studies show that aperiodic tilings can serve not only as mathematical descriptions of non-periodic order but also as generative frameworks for structural topology and mechanically functional lattice systems.

The present study deliberately departs from the original mathematical formulation of the Ammann A4 set. Rather than reconstructing a mathematically valid aperiodic tiling governed by the complete matching conditions and substitution hierarchy, the geometries of the A- and B-prototiles and the associated Ammann-line organisation are treated as generative references for periodic two-layer structural configurations. The resulting systems should therefore be understood as Ammann-inspired periodic structures rather than as mathematical instances of the original aperiodic Ammann A4 tiling.

### 1.1. Research Gap

Aperiodic tilings have been extensively studied as mathematical systems in which local matching rules generate long-range non-periodic order. In architectural and structural contexts, tiling systems have also been investigated as sources of geometric organisation, spatial subdivision, and non-standard lattice configurations [1,3]. However, the structural studies identified above have predominantly considered aperiodic patterns as complete lattice or cellular topologies. A different question arises when selected geometric features of an aperiodic tiling are detached from their original matching-rule context and reassigned distinct mechanical roles within a periodic spatial structural system. In particular, the literature considered here does not address the use of Ammann A4 geometry to generate repeatable two-layer configurations in which the tile-edge network, Ammann-line subsystem, and interlayer diagonals are deliberately differentiated according to structural function [2,6,9,11,14,15,16,17,18].

Mechanical studies of aperiodic lattices further indicate that the consequences of aperiodic organisation extend beyond geometric novelty. Moat et al. [17] demonstrated differences in directional mechanical response and strain localisation in cellular structures derived from Penrose P3 tilings, while Imediegwu et al. [12] showed that the ordered but non-periodic distribution of member orientations and connectivity can produce near-isotropic mechanical behaviour. The recent results of Qi et al. further demonstrate that different Penrose-derived topological configurations can produce distinct deformation and failure mechanisms [13]. Collectively, these findings indicate that structural response in tiling-derived systems depends not merely on the presence or absence of periodicity, but on the specific topology, connectivity, and directional organisation generated by the underlying pattern. Related studies of aperiodic honeycombs further show that tiling geometry can be exploited to control properties such as isotropy, Poisson’s ratio, and stiffness [19]. Collectively, these findings indicate that structural response in tiling-derived systems depends not merely on the presence or absence of periodicity, but on the specific topology, connectivity, and directional organisation generated by the underlying pattern.

The mechanical behaviour of such systems nevertheless depends strongly on the particular geometric organisation from which the lattice is generated. Somera et al. demonstrated that quasi-periodic lattices may exhibit stretching-dominated, bending-dominated, or intermediate behaviour depending on the elementary pattern geometry [17]. More recently, Clarke et al. addressed the additional difficulty of defining mechanically representative domains for aperiodic honeycombs [20,21], for which the absence of translational symmetry prevents the straightforward use of a conventional periodic unit cell. Their numerical and experimental results showed how the size of the sampled aperiodic domain affects the convergence and variability of predicted mechanical properties. Rosa et al. further showed, for the quasicrystalline architected materials examined in their study, that aperiodic beam networks can redistribute force paths and promote spatially distributed local buckling rather than the more global instability modes observed in their periodic reference architectures [22]. It should be emphasised, however, that these studies primarily investigate the in-plane mechanical response of tiling-derived cellular systems, whereas in the present study the external loading acts transverse to the plane of the tiling-derived geometry and is transferred through a two-layer spatial system, constituting a fundamentally different structural application of tiling-based topology.

Ammann-line constructions provide an additional geometric route from aperiodic tiling theory toward structural organisation. Tarczewski considered Ammann bars as a potential geometric basis for spatial structural lattices and discussed their relationship to plate-lattice dualism [5]. This interpretation is particularly relevant to the present study because continuous Ammann lines can be regarded not only as auxiliary constructions associated with aperiodic order, but also as potential geometric trajectories for mechanically differentiated structural members. The present work develops this possibility in a different direction: rather than constructing an aperiodic lattice directly from a tiling, selected geometric relationships associated with Ammann A4 prototiles and Ammann-line organisation are reformulated into repeatable two-layer spatial systems in which the upper, lower, and diagonal member groups are assigned distinct structural roles.

The research gap addressed in this study lies at the intersection of aperiodic tiling theory and structural design methodology. While the original Ammann A4 system is governed by matching conditions that generate aperiodic order, practical spatial structures require different attributes. They demand controllable connectivity, repeatable geometric organization, explicit support conditions, and a clear mechanical interpretation of individual member groups.

To bridge this gap, the present study investigates whether selected geometric principles derived from the Ammann A4 system can be deliberately detached from their original aperiodic context. These principles are subsequently reformulated as periodic two-layer structural configurations suitable for comparative geometric and numerical evaluation. Specifically, we utilize Ammann lines—which originally served as an auxiliary mathematical construction ensuring aperiodicity—as the generator for a completely new concept of the grid’s bottom layer.

### 1.2. Study Objectives and Innovativeness

The objective of this study is to develop and comparatively evaluate periodic two-layer spatial configurations inspired by the geometry of the Ammann A4 prototiles and the associated Ammann-line system. The study does not aim to reproduce a mathematically valid Ammann A4 tiling or to demonstrate aperiodicity. Instead, selected geometric relationships derived from the A- and B-prototiles are used as a generative framework for structural organisation. The resulting configurations combine an upper tile-derived grid, a lower line-based subsystem intended to act predominantly in tension, and diagonal members coupling the two layers. Their structural behaviour is subsequently evaluated through finite element analysis under common assumptions regarding material properties, member cross-sections, loading, and support rules.

The principal innovative aspects of the study are:

- The reinterpretation of Ammann A4 geometry from a mathematical tiling system into a generative framework for periodic two-layer structural configurations;
- The explicit distinction between the original aperiodic Ammann A4 tiling and the periodic Ammann-inspired structural configurations developed in the study;
- The introduction of a two-layer structural interpretation in which the tile-derived upper grid, the continuous Ammann-line-based lower subsystem, and the diagonal interlayer connections are treated as geometrically related but mechanically distinct components;
- The formulation of a rule-based procedure for positioning diagonal members, allowing interlayer connections to be generated according to repeatable geometric criteria rather than by arbitrary placement;
- The comparative evaluation of three newly developed Ammann-inspired configurations against both an Ammann A4-based reference configuration and the classical Unibat double-layer grid [23,24,25].

### 1.3. Translating Tiling Geometry into Structural Form

In translating tiling geometry into structural form, the prototiles are not treated as surface units to be reproduced literally, but as geometric elements that define the topology of the structural system. Their boundaries establish the upper-layer network, while their adjacency relationships determine the distribution of nodes and potential member connections [26,27]. A second, line-based organisation derived from the Ammann-bar system is introduced below the upper layer as the basis of the lower subsystem, whose structural response is subsequently examined through numerical analysis.

The resulting spatial system therefore comprises three interdependent structural components:

- An upper layer defined by the boundaries of the A- and B-prototile arrangement;
- A lower, line-based subsystem derived from selected continuous Ammann lines and intended to act predominantly in tension;
- Diagonal members that connect the two layers and enable force transfer between them.

These components are geometrically related but mechanically distinct, and their interaction forms the basis of the structural configurations analysed in this study.

The research therefore focuses on the structural consequences of this geometric translation rather than on the mathematical completeness of the original tiling rules. Of particular importance is the continuity of the lower line-based subsystem. Because this subsystem is intended to function predominantly in tension, the selected Ammann lines must form uninterrupted and directionally continuous paths between supports. Their continuity, directionality, and support-to-support extension are therefore treated as explicit design criteria, allowing the tiling-derived linear order to function as a structurally meaningful framework rather than merely as a geometric pattern.

### 1.4. Analysed Patterns

**Figure 3:**
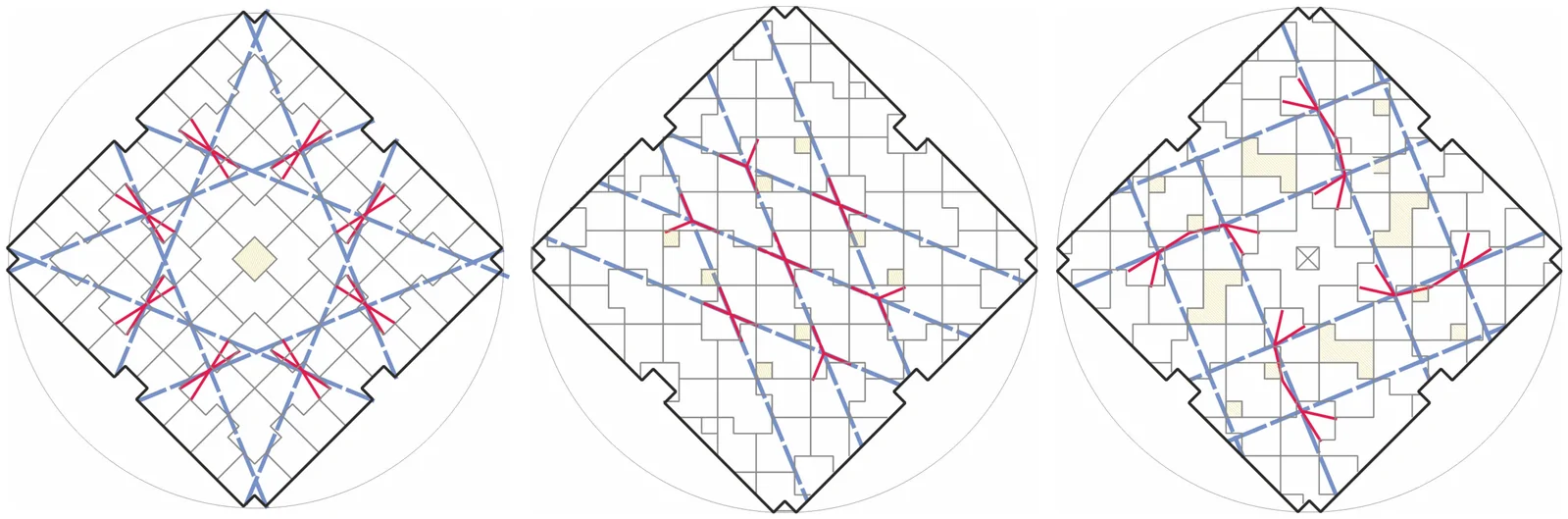
Three Ammann-inspired periodic structural configurations (V1–V3) analysed in the study. The upper layer is derived from arrangements of A- and B-tiles, while the blue lines indicate selected continuous paths in the lower tensile subsystem based on Ammann lines. Red lines represent the diagonal elements that couple the upper and lower layers. The variants are ordered from left to right by increasing relative heterogeneity. These models should be understood as periodic structural configurations inspired by Ammann tile geometry rather than as mathematical instances of the original aperiodic Ammann A4 tiling. The outer boundary line in thick black represents the edge of the structural system. Yellow shading indicates residual polygonal regions.

Within this framework, three periodic structural configurations, designated V1, V2, and V3, were developed for comparative analysis. Each follows the same general organisational principle: an upper layer derived from arrangements of A- and B-prototiles, a lower line-based subsystem organised by selected continuous Ammann lines, and diagonal members connecting the two layers. The configurations differ in tile arrangement, member density, connectivity, and the spatial distribution of interlayer connections, as shown in Figure 3. For descriptive purposes, V1–V3 are ordered by increasing relative heterogeneity, defined here as the number of residual polygonal regions divided by the total number of A-tiles, B-tiles, and residual polygonal regions within the analysed structural field. This parameter is used only as a geometric ordering descriptor and not as an independent explanatory variable, because several geometric and topological characteristics vary simultaneously among the three configurations.

Configurations V1–V3 were developed through a manual, exploratory design process rather than a predefined generative algorithm. Tiles A and B were progressively assembled and rearranged, with the continuity of all Ammann lines checked throughout each resulting tiling. This continuity requirement served as a geometric acceptance criterion, while the arrangement of the tiles remained a design choice. Complete coverage of the layout by tiles A and B was not imposed as a design requirement. The manual exploration therefore allowed residual polygonal regions between the assembled tiles. These regions were not introduced as additional predefined tile types; their shapes and sizes emerged from the arrangement of tiles A and B. Their presence was accepted provided that the continuity of all Ammann lines was maintained throughout the layout, including across the residual regions. The resulting configurations thus combine arrangements of tiles A and B with intervening polygonal regions, rather than constituting complete tilings formed exclusively by these two tile types.

A subsequent selection step determined which Ammann lines were used to define the lower chords of the structural configurations. The principal selection criterion was that these lines should traverse the layout between opposite regions of its boundary, following approximately diametral paths. Lines that crossed only part of the layout without connecting opposite boundary regions were not selected. This was a qualitative geometric criterion applied by the designer, rather than a numerical selection rule. V1–V3 represent three examples obtained through this exploratory process, rather than an exhaustive or optimized set of configurations; other arrangements satisfying the same continuity requirement and selection criterion are possible.

The Ammann lines selected to define the lower chords intersect at multiple locations; however, structural joints are provided only at those intersections where diagonal members connect the lower and upper layers. All other crossings are geometric intersections without a structural connection between the intersecting lower-chord members.

Accordingly, V1–V3 are treated as complete structural configurations developed under a common set of design constraints rather than as outcomes of a one-variable parametric experiment. This approach enables comparison of how different periodic arrangements inspired by Ammann geometry influence structural organisation and the resulting numerical response.

## 2. Geometric and Structural Methodology

The methodology was organised as a two-stage procedure combining geometric construction with structural modelling:

- **Stage 1**—Geometric construction: Three periodic Ammann-inspired configurations, V1–V3, were developed as spatial geometric models based on the arrangement of A- and B-prototiles, the definition of the upper layer, the selection of continuous Ammann-line paths for the lower subsystem, and the placement of diagonal members connecting the two layers. In parallel, two reference configurations were prepared: one based on the original Ammann A4 tessellation and one corresponding to the classical Unibat double-layer grid.
- **Stage 2**—Structural modelling: All five configurations were translated into node-and-member models suitable for finite element analysis. Their geometric relationships were converted into explicit structural connectivity, member groups, and boundary conditions, providing a consistent basis for comparative numerical evaluation.

This separation allowed the geometric logic of each configuration to be described independently from its subsequent structural interpretation and numerical analysis.

### 2.1. Geometric Construction of the Analysed Configurations

The geometric construction began with periodic arrangements derived from the geometries of the Ammann A- and B-prototiles. These arrangements were not intended to reproduce the original aperiodic A4 tiling, but to generate repeatable geometric fields suitable for translation into two-layer spatial structures. In each configuration, the prototile boundaries defined the upper structural layer, while selected continuous paths derived from the Ammann-line system formed the basis of the lower subsystem.

The two layers were subsequently connected by diagonal members placed at geometrically defined locations. Because the tile arrangement, continuity of the lower subsystem, distribution of interlayer connections, and relative heterogeneity were mutually dependent, these characteristics could not be varied independently during the development of the configurations. The design procedure therefore followed a rule-constrained manual process based on the following requirements:

- The correspondence between the prototile arrangement, the Ammann-line network [2,9,28], and the spatial positioning of the diagonal members was established manually. Although the procedure was not automated, it was governed by explicit geometric rules and subsequently documented using measurable geometric and topological descriptors for each configuration.
- A further selection criterion concerned the mechanical continuity of the intended tensile subsystem. Each selected Ammann line was required to form an uninterrupted and directionally continuous path between boundary supports. Configurations in which a presumed tensile path changed direction at an angular junction were rejected, because a tensile member cannot sustain an abrupt change in direction without an intermediate deviator, saddle, or another connection capable of balancing the resulting transverse force. Intersections between distinct Ammann lines were therefore treated as crossings or separate structural nodes rather than as bends within a single tensile path.

### 2.2. The Diagonal Struts

The diagonal struts were introduced at selected nodes of the lower line-based subsystem to provide structural coupling between the upper and lower layers while preserving the directional organisation of the Ammann-inspired geometry. Only a subset of the intersections within the lower Ammann-inspired line network was used as nodes (structural joints) for diagonal members. The design intent was to distribute the interlayer connections across the roof area, providing support to the upper grid as evenly as practicable within the adopted geometry while preserving the symmetry of each configuration. Also, the node placement follows the symmetry of each configuration, including the rotational symmetry of corresponding groups. This was a qualitative design objective rather than a numerical uniformity criterion or an optimisation procedure. Once the lower connection nodes had been chosen, the circle-based construction described below determined their connections to the upper-layer nodes. Alternative selections of lower connection points were not investigated; consequently, the reported results characterise the adopted configurations, and the influence of connection-point selection was not assessed independently.

As above described, the diagonal struts were introduced at selected nodes (structural joints) of the lower line-based subsystem to provide structural coupling between the upper and lower layers while preserving the directional organisation of the Ammann-inspired geometry. Their locations were determined by a repeatable geometric procedure rather than by arbitrary placement.

The diagonal members were generated according to the following three-step rule:

- Intersection points within the lower Ammann-inspired line network were identified as potential lower-layer nodes (structural joints);
- For each node (structural joint), a circle was constructed in plan, centred at that point, with a radius equal to the distance to the nearest node of the upper tile-derived layer;
- Diagonal members were introduced between the lower-layer node and the upper-layer nodes lying on the circumference of the corresponding circle.

Because the vertical separation between the two layers was constant for the relevant nodes, diagonal members generated from the same plan radius had identical lengths and inclination angles. The procedure therefore provided a geometrically standardised method for defining interlayer connections and reduced arbitrariness in their placement.

The diagonal system was not intended to form a uniform space-frame network. Instead, the connections occurred at selected intersection nodes of the lower subsystem and at geometrically corresponding nodes of the upper grid. Consequently, their number and spatial distribution varied among the configurations and reflected the geometry of each arrangement. The resulting diagonal members provided the interlayer connectivity required for the upper and lower subsystems to participate in a common structural response under vertical loading.

### 2.3. Variant Description

The three principal configurations, V1–V3, should be interpreted as distinct organisational states generated within the same Ammann-inspired design framework rather than merely as successive levels of relative heterogeneity (Figure 3):

1. **Variant V1** represents the most spatially continuous and evenly distributed configuration, with a dense upper-layer organisation and continuous lower-system paths distributed throughout the structural field.
2. **Variant V2** exhibits a more pronounced directional organisation, with selected linear paths and interlayer connections becoming more prominent within the overall configuration.
3. **Variant V3** combines the highest relative heterogeneity with a more selective distribution of structural components and locally concentrated connections between the upper and lower subsystems.

Accordingly, the sequence V1–V3 represents a transition from a relatively continuous and distributed organisation toward configurations with increasingly directional and selective structural connectivity. Relative heterogeneity is used here only as one descriptive geometric characteristic and not as an independent variable controlling this transition.

**Figure 4:**
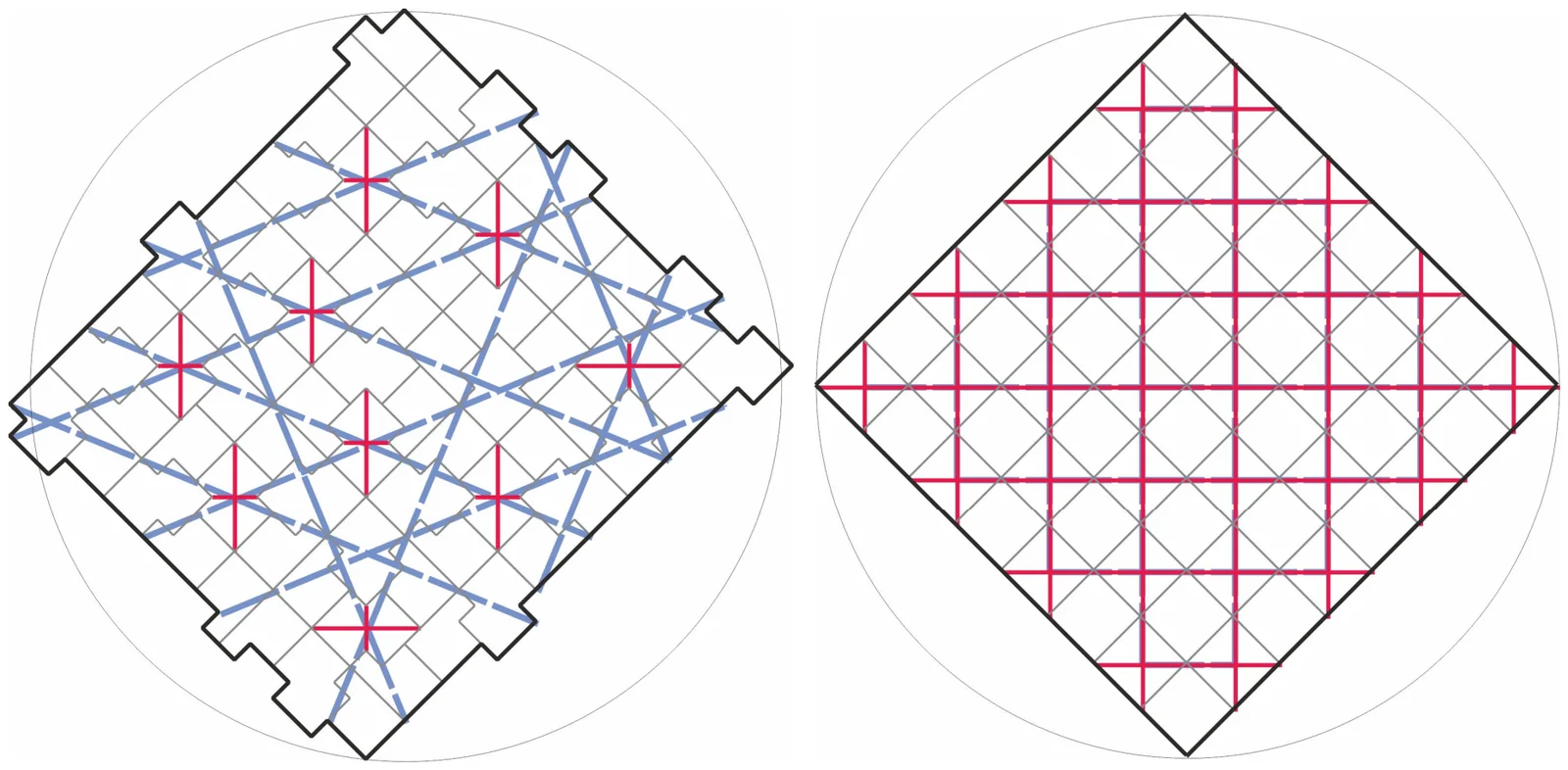
Two auxiliary variants investigated as a comparative baseline for variants V1–V3: (**left**) the variant based on the Ammann A4 aperiodic tessellation, where the upper layer is derived from the edges of tiles A and B, and the lower layer is aligned with the Ammann bars of this tessellation; (**right**) the variant based on Stéphane DuChâteau’s topology for the Unibat double-layer grid. Graphical notations are consistent with Figure 3.

To provide comparative reference points for the three newly developed configurations, two additional structural systems were analysed, as shown in Figure 4:

1. **Ammann A4 reference configuration.** The upper layer was derived from an aperiodic Ammann A4 tessellation using the same general structural interpretation adopted for V1–V3. The lower subsystem consisted of members aligned with the Ammann bars of the corresponding tiling. Diagonal struts were positioned according to the same general geometric principles used for the three periodic variants, and lower-layer members not connected to diagonal struts were omitted from the structural model. Unlike V1–V3, whose overall boundaries approximate a square inscribed within the common reference circle, the aperiodic organisation of the Ammann A4 tessellation produces an irregular perimeter with local outward projections beyond the reference square.
2. **Unibat reference configuration.** The second reference model reproduced the topology of the classical Unibat double-layer grid developed by Stéphane DuChâteau [23,25]. Its upper layer consists of an orthogonal square grid, while the lower-layer network is rotated by 45° relative to it. Lower-layer intersection nodes are positioned beneath the centres of alternating upper-grid squares, producing the characteristic checkerboard arrangement. These nodes are connected to the upper layer by diagonal members forming repetitive pyramidal units. In contrast to the Ammann-inspired configurations, the Unibat model has a regular square perimeter inscribed within the same reference circle.

In all five configurations, the upper structural layer is defined by the primary geometric network of the system, whereas the lower subsystem is derived from a secondary line-based organisation. In the Ammann-inspired configurations, this lower network follows selected Ammann-line paths, while in the Unibat reference it follows the regular rotated lower grid characteristic of that system. The upper and lower layers are connected by diagonal members that provide structural coupling between the two subsystems. For variants V1–V3 and the Ammann A4 reference, the lower subsystem is intended to act predominantly in tension; this mechanical role is treated here as a design hypothesis and is subsequently verified through finite element analysis. The numbers and distributions of nodes, members, and supports, together with the principal geometric descriptors of all five configurations, are summarised in Table 1. Interactive three-dimensional representations of the analysed systems are provided in Models S1–S5.

### 2.4. Structural Analysis

The structural behaviour of the five configurations was evaluated within a comparative finite element modelling framework. Each system was represented as a three-dimensional member model. To maximise comparability, common assumptions were adopted for material properties, the vertical separation of the principal layers, member cross-sections assigned to corresponding structural subsystems, and loading. The geometric topology of each configuration was retained, however, so that differences in structural response could be examined in relation to the organisation of the individual systems rather than to differences in basic modelling assumptions.

Following the geometric construction stage, each configuration was translated into an explicit node-and-member structural model [18,25]. The connectivity of the system was therefore defined not merely by the geometric intersection of lines but by shared structural nodes connecting the corresponding members. Members were classified as belonging to the upper layer, the lower layer, or the diagonal subsystem, allowing the mechanical response of these groups to be evaluated separately in the subsequent analysis.

To perform the comparative computational analysis, numerical models of all five configurations were developed in Autodesk Robot Structural Analysis Professional (ARSAP) 2014.

The numerical model in ARSAP employs beam elements with uniform cross-sections and no offsets. Because no end releases were applied, the elements function as fixed-fixed members. In the top layer and for the diagonal struts, every geometric node serves as a structural node for the converging members. In the bottom layer, member axis intersections are treated as structural nodes only where they connect to the diagonal struts; elsewhere, the members cross without interaction. The nodal connections are assumed to be rigid, with no defined flexibility, with the exception of the support nodes.

Each member is assigned a right-handed Cartesian local coordinate system (*x*, *y*, *z*), following the standard ARSAP convention. The local *x*-axis runs along the member’s longitudinal axis, directed from the start node to the end node. The local *y*-axis is parallel to the global *XY* plane; it is defined by a vector perpendicular to both the local *x*-axis and the global *Z*-axis, oriented such that its projection onto the global *Y*-axis (or the *X*-axis where applicable) is positive. The local *z*-axis completes the right-handed system, lies within the vertical plane passing through the member, and has a positive projection onto the global *Z*-axis.

Two load cases were defined. The first included the self-weight of all the structural members, while the second involved a uniformly distributed vertical load applied to the upper layer. This surface load was transferred to the top layer using a two-way cladding object. It was distributed to the individual members of the layer using the envelope method (tributary area method, involving triangular and trapezoidal distribution zones). Consequently, each perimeter or internal member received a line load (varying as a trapezoid/triangle or remaining constant) proportional to its geometrically assigned tributary area. This represented a purely geometric load distribution that accounted for neither the stiffness of the members themselves nor the multi-span stiffness and continuity of the cladding. The total design loads applied to the analysed configurations are presented in Table 1.

No tension-only or compression-only behavior was imposed on any member group; all members were permitted to develop both tensile and compressive axial forces. This assumption was necessary because the predominantly tensile role attributed to the lower subsystem constituted a design hypothesis to be tested by the numerical analysis rather than a predefined mechanical constraint.

The support conditions were defined according to a common boundary principle adapted to the topology of each configuration. Because the five systems differ in topology and perimeter geometry, the exact number and spatial distribution of supports were not identical. In the Ammann-inspired variants, supports were assigned to all nodes along the top-layer perimeter and to the end nodes of bottom-layer members terminating at the boundary.

In the Unibat reference configuration, supports were located exclusively in the top layer, specifically at all perimeter nodes, while no supports were applied to the bottom-layer nodes. This aligns with the classical arrangement of supports in structures of this type, thereby serving as a reference to the traditional geometries of double-layer grids.

These assumptions led to variations in the total number of supports across the models, particularly for the Unibat variant. Such differences are treated as inherent characteristics of the complete configurations and are explicitly documented in Table 1. However, the general premise was to provide boundary constraints along all edges using a discrete support arrangement. Consequently, despite the differences in support counts, the boundary conditions across all variants remained consistent enough to enable a reliable comparison of the computational results.

## 3. Computational Analysis Flow

The physical dimensions assigned to the computational models of the structures were chosen so that each configuration fit within the same reference circle, with a diameter D = 63.176 m, as illustrated in Figure 3 and Figure 4. The corresponding plan dimensions of the models were 46.627 × 46.627 m. These non-standard dimensions resulted from the scaling procedure adopted during model development, in which the overall geometry was adjusted so that the baseline member length in variants V1–V3 did not exceed approximately 5.0 m. The resulting baseline upper-layer member length was 4.828 m. Owing to their different geometric organizations, the corresponding baseline member length was 4.579 m in the Ammann A4 reference configuration. In contrast, for the Unibat reference configuration, the priority was to maintain a similar number of perimeter members to the other variants, rather than to match the baseline member length; this approach yielded a resultant length of 5.584 m. The principal geometric and topological descriptors of all five analysed configurations are summarized in Table 1.

The vertical separation between the principal upper and lower layers was generally 4.00 m. In variants V1–V3 and Ammann A4, however, the lower-layer boundary nodes connected to supports were positioned only 1.00 m below the upper layer, whereas in the Unibat reference configuration, the corresponding boundary nodes of the lower layer retained the full 4.00 m offset. This is because the Unibat configuration differed from the Ammann-inspired systems in that all its supports were located exclusively in the upper layer. These upper-layer boundary supports in all configurations restrained vertical translation (*U_z_*) while allowing for horizontal translations and rotations. To prevent rigid-body motion and ensure global model stability, two additional in-plane constraints were introduced at nodes located on opposite sides of the structure: one node was restrained in the y-direction (*U_y_*), while the other was restrained in both the x- and y-directions (*U_x_*, *U_y_*). All lower-layer supports in the remaining variants were modeled as fully pinned, restraining translations in *U_x_*, *U_y_*, and *U_z_* while allowing rotations.

To maximize comparability among the configurations, identical material properties and member cross-sections were assigned to the corresponding structural subsystems in all five models. All members were modeled in S355 structural steel. The upper-layer members were assigned SHS 200 × 10 profiles, the lower-subsystem members CHS 101.5 × 8 profiles, and the diagonal members SHS 80 × 8 profiles. Keeping these properties constant ensured that differences in the calculated structural response were associated primarily with the geometry and topology of the configurations rather than with variant-specific differences in member stiffness or material properties.

Two load cases were applied to each structural model:

- **Self-weight (STA1):** The self-weight of all structural members was generated automatically by the software on the basis of the assigned material properties and member cross-sections;
- **Imposed load (EKSP1):** A uniformly distributed vertical surface load of 0.50 kN/m^2^ was applied to the entire upper structural layer in the negative global *z*-direction. This surface load was converted by the software into equivalent line loads acting on the corresponding upper-layer members, as described in Section 2.4.

A uniform surface load was adopted to provide a consistent loading condition for configurations with different upper-layer topologies. Applying concentrated loads at selected nodes would have made the direct comparison more dependent on the local geometry and nodal arrangement of each variant. The imposed load magnitude was selected for comparative analysis rather than for the design verification of a specific building application. Member cross-section optimization, detailed strength verification, stability assessment, and serviceability checks therefore fell outside the scope of the present study. Consequently, comparing the stress levels of the members and the structural mass yields limited utility; nevertheless, the latter parameter is presented in Table 1.

The two load cases were combined using unit load factors as (KOMB1 = STA1 + EKSP1). All axial forces, bending moments, and nodal displacements discussed in the following sections correspond to this load combination.

The numerical simulations in ARSAP were performed using a geometrically nonlinear analysis accounting for P-Delta effects. The solution for the nonlinear system of equations was obtained iteratively using the standard Newton–Raphson method available in the software. By employing this advanced computational approach, the structural behavior under the combined loading was captured with high precision, establishing a rigorous foundation for the comparative evaluation of the configurations presented in the following section.

## 4. Results of Computational Analysis

Computational analyses were performed for all five structural configurations described above. Although detailed results for the Ammann and Unibat configurations are provided in the Supplementary Materials, these variants are included in the comparative interpretation of the results. The analysis is organised by structural response rather than by individual configuration. Accordingly, axial-force (*F_x_*) distributions are first compared across variants V1, V2, and V3, followed by the total resultant moment magnitude (*M_r_*) and, finally, vertical nodal displacements (*U_z_*). OpenAI’s Data Analyst tool was used to assist with processing and presenting the exported numerical results. However, the AI-assisted statistical analyses and visualisations were fully reviewed and verified, and full responsibility is taken for the reported methods and results. The structural geometries and finite element models were independently developed, and all structural calculations were performed using Autodesk Robot Structural Analysis Professional.

The quantity *M_r_* represents the resultant of the bending moments acting on the member about the local *Y* and *Z* axes. It is calculated as the square root of the sum of the squares of the local components, *M_y_* and *M_z_*. Since the moment *M_x_* represents the torsional moment in the local coordinate system, it is excluded from the *M_r_* calculation; its detailed descriptive statistics are instead provided in Table S4 in the Supplementary Materials. Furthermore, to account for differences in member length, the resultant moments were normalised by dividing each *i*-member value by its length: *M_rn_*_,*i*_ = *M_r_*_,*i*_/*L_i_*, expressed in kNm/m. The corresponding descriptive statistics are provided in Table S3 in the Supplementary Materials. This normalisation is distinct from calculating length-weighted averages; each member has equal weight in the descriptive statistics.

Organising the results by response quantity facilitates direct comparison using common metrics and makes it possible to identify differences in force distribution, deformation patterns, and load-transfer mechanisms while retaining the limitations arising from the topological and support-system differences among the configurations.

The purpose of the analysis is not to develop code-compliant designs for the individual variants but to compare, under common loading, material, and member-section assumptions, the structural responses of three newly developed periodic Ammann-inspired variants V1 through V3, in which the lower subsystems are derived from Ammann lines and are intended to act predominantly in tension. The Ammann A4 and Unibat configurations serve as reference configurations. The member cross-sections were therefore kept unchanged and were not optimised separately to satisfy design-code stress or displacement limits, because variant-specific sizing would introduce additional variables and obscure differences associated with geometric organisation. Consequently, the analysis focuses on relative patterns of axial-force distribution, total resultant moment magnitude, and vertical displacement rather than on ultimate load-bearing capacity or serviceability compliance.

**Figure 5:**
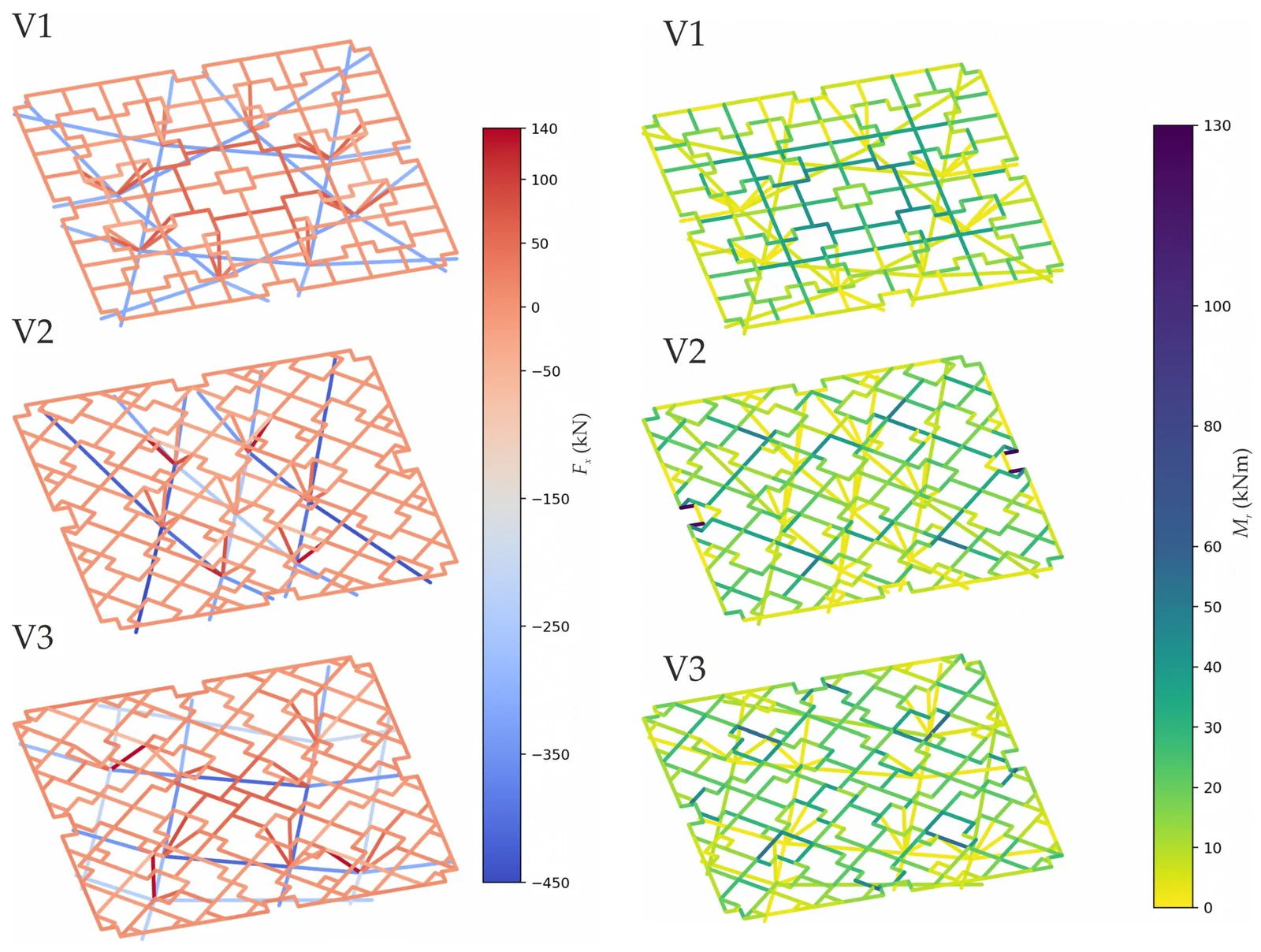
Comparative structural responses of variants V1–V3: axial forces (*F_x_*) (**left**) and bending moments (*M_r_*) (**right**). Colours represent the corresponding values according to the adjacent scales, expressed in kN and kNm, respectively. Identical viewpoints and common colour-scale ranges are used for each response parameter to enable direct comparison among the three configurations.

**Figure 6:**
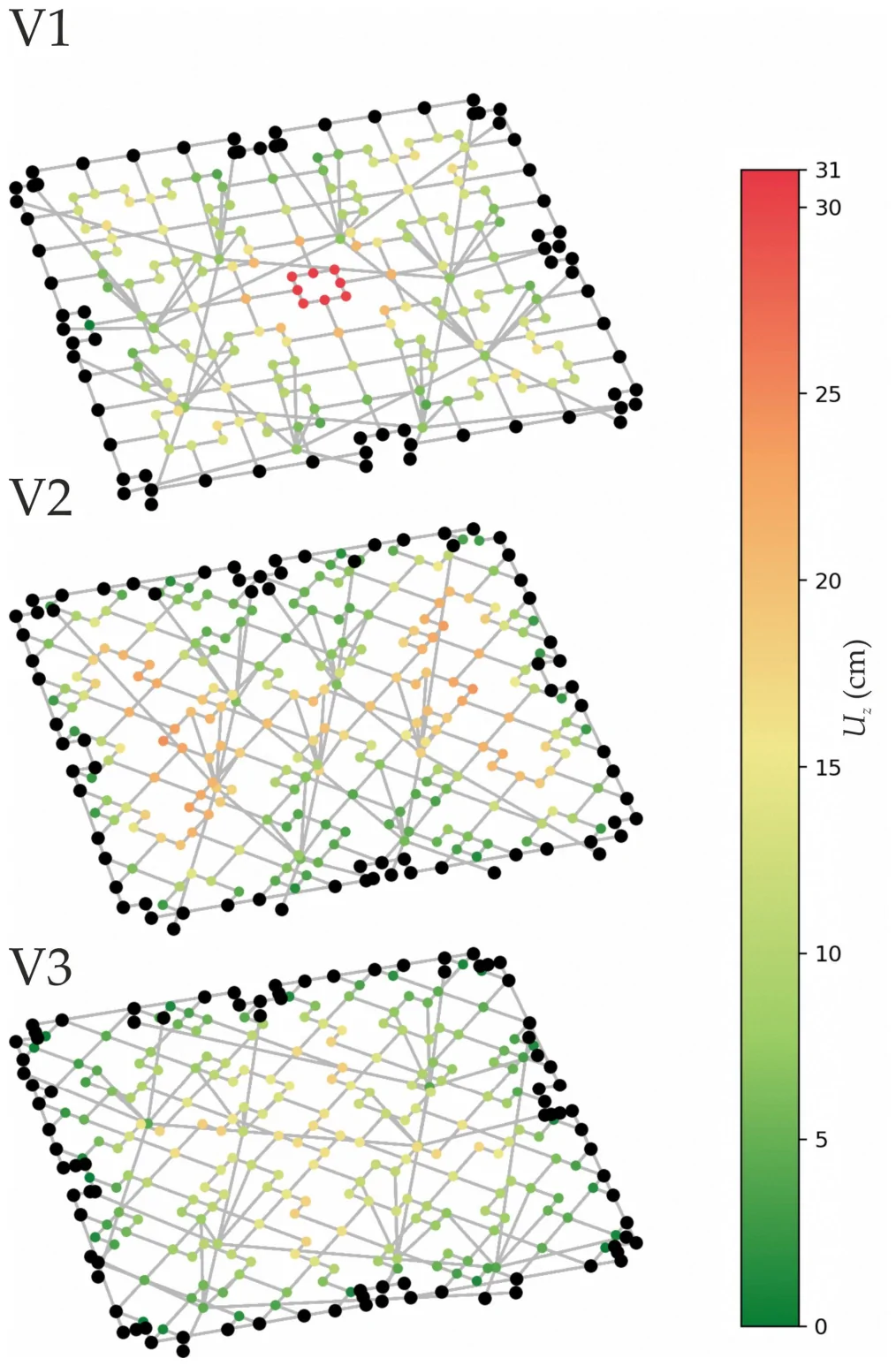
Comparative structural responses of variants V1–V3: vertical nodal displacements (*U_z_*). Coloured nodes represent vertical displacement values (cm), according to the common colour scale. Grey lines indicate structural members, while black dots identify support nodes. Identical viewpoints and a common colour scale are used to facilitate direct comparison between the three variants.

Figure 5 and Figure 6 provide graphical comparisons of the structural responses for variants V1–V3, while Figures S1 and S2 show those for the Ammann and Unibat variants; the figure captions specify the visualisation conventions and scale ranges.

All reported standard deviations are population standard deviations calculated over the analysed set of members or nodes. Coefficients of variation for lower-subsystem axial forces are calculated as the standard deviation of the member force magnitudes divided by their mean magnitude. Displacement statistics include all nodes in the relevant configuration or nodal layer, including recorded zero values. No separate free-node statistics are used.

### 4.1. Axial Force (F_x_) Distributions

Axial-force distributions are presented using the sign convention adopted in Robot (ARSAP), where positive (*F_x_*) values denote compression and negative values denote tension. For the statistical comparisons, each member is represented by the signed endpoint value with the greatest absolute magnitude. Figure 5 shows the distributions for variants V1–V3 using a common colour scale, enabling a direct comparison of both the magnitude and spatial distribution of the axial forces.

- Variant V1 exhibited a clear separation of axial-force functions among the three member groups. The upper grid carried comparatively small and mixed axial forces, ranging from −40.03 to +61.69 kN, with a mean of −1.47 kN. All 24 members of the lower subsystem were in tension, with *F_x_* values ranging from −324.68 to −291.79 kN and a mean of −308.58 kN. The limited standard deviation of 12.21 kN, corresponding to a coefficient of variation of 4.0%, indicates that V1 provided the most uniform activation of the lower tensile subsystem among variants V1–V3. All 32 diagonal members were in compression, with forces ranging from +5.08 to +71.98 kN and a mean of +46.79 kN. The maximum absolute axial force was 324.68 kN and occurred in the lower subsystem; this value was lower than the corresponding extremes recorded for V2 and V3.
- Variant V2 exhibited low and mixed axial forces in the upper grid, whereas substantially greater forces occurred in the lower subsystem and diagonal members. Across the 360 upper-grid members, *F_x_* ranged from −73.10 to +14.99 kN, with a mean of −4.51 kN. All 20 members of the lower subsystem were in tension, with forces ranging from −448.32 to −243.54 kN and a mean of −350.92 kN. The corresponding standard deviation of 67.31 kN and coefficient of variation of 19.2% indicate a less uniform tensile-force distribution than in V1 but a more uniform distribution than in V3. The diagonal system was predominantly in compression: 22 of its 24 members were in compression, and 2 were in tension, with forces ranging from −3.25 to +133.01 kN and a mean of +48.58 kN. The maximum absolute axial force of 448.32 kN occurred in the lower subsystem and was the highest value recorded among variants V1–V3. V2 therefore combined the greatest tensile demand in the lower subsystem with an intermediate degree of force uniformity.
- Variant V3 exhibited mixed but comparatively moderate axial forces in the upper grid, with *F_x_* ranging from −64.59 to +75.33 kN and a mean of +3.86 kN. All 24 members of the lower subsystem were in tension, with forces ranging from −412.46 to −193.84 kN and a mean of −292.04 kN. The corresponding standard deviation of 70.09 kN and coefficient of variation of 24.0% indicate the greatest relative variability in the lower tensile subsystem among variants V1–V3. The diagonal system was predominantly in compression: 20 of its 24 members were in compression, and 4 were in tension, with forces ranging from −19.13 to +144.74 kN and a mean of +57.15 kN. The greatest tensile force magnitude, |*F_x_*| = 412.46 kN, occurred in the lower subsystem and was greater than that recorded for V1 but lower than that recorded for V2. V3 therefore combined the highest diagonal compressive force with the least uniform activation of the lower tensile subsystem among the three variants.

Compared with the Ammann A4 and Unibat reference configurations, variants V1–V3 exhibited more uniform axial-force magnitudes in their lower subsystems. The corresponding coefficients of variation were 4.0% for V1, 19.2% for V2, and 24.0% for V3, compared with 38.6% for the Ammann reference and 40.7% for Unibat. The greatest tensile forces in V1 and V3 (*F_x_* = −324.68 and −412.46 kN, respectively) were lower in magnitude than that recorded for the Ammann reference (*F_x_* = −417.76 kN), whereas V2 exceeded it, reaching −448.32 kN. The Unibat reference exhibited a different response: its greatest tensile force was −186.87 kN, but it developed the highest compressive force of all five configurations, reaching +184.31 kN, and the highest mean absolute member force, at 72.94 kN. Its diagonal system also showed an almost balanced tensile–compressive response, unlike the predominantly compressive diagonal action observed in the Ammann reference and variants V1–V3. Because the configurations differ in topology and member count, these results should be interpreted as comparative response descriptors rather than direct measures of structural efficiency.

### 4.2. Resultant Moment Magnitude (M_r_) Distributions

Figure 5 presents resultant moment magnitudes using a common colour scale. For the statistical comparisons, each member is represented by the maximum *M_r_* value recorded at either endpoint. Because the reported *M_r_* values are non-negative, the comparison concerns their magnitude, dispersion, and spatial concentration rather than their sign. The results are also examined separately for the upper grid, lower subsystem, and diagonal members to determine how the reported moment response is distributed among the principal structural components.

- For V1, the maximum member values of *M_r_* ranged from 1.33 to 72.57 kNm, with a mean of 25.62 kNm, a median of 21.40 kNm, and a standard deviation of 19.77 kNm. The global maximum of 72.57 kNm occurred in the upper grid, in member 283 at node 219. The mean value for the upper grid was 30.06 kNm, compared with 5.82 kNm for the lower subsystem and 2.76 kNm for the diagonal members; the corresponding maxima were 72.57, 7.76, and 3.92 kNm. Of the 328 members, 58 (17.7%) reached *M_r_* ≥ 50 kNm, whereas none reached 100 kNm. These results indicate that the highest *M_r_* values and the largest group mean occurred in the upper grid, while the lower subsystem and diagonal members exhibited substantially smaller values.
- For V2, the maximum member values of *M_r_* ranged from 1.35 to 129.12 kNm, with a mean of 24.33 kNm, a median of 21.91 kNm, and a standard deviation of 20.04 kNm. The global maximum of 129.12 kNm occurred in the upper grid, in member 356 at node 226. The mean value for the upper grid was 26.74 kNm, compared with 5.55 kNm for the lower subsystem and 3.77 kNm for the diagonal members; the corresponding maxima were 129.12, 13.84, and 4.52 kNm. Of the 404 members, 38 (9.4%) reached *M_r_* ≥ 50 kNm, including 4 (1.0%) with *M_r_* ≥ 100 kNm. Thus, although the overall mean was lower than in V1, the maximum was approximately 78% higher, indicating a more localised concentration of high *M_r_* values in the upper grid.
- For V3, the maximum member values of *M_r_* ranged from 0.49 to 90.76 kNm, with a mean of 26.37 kNm, a median of 24.01 kNm, and a standard deviation of 18.58 kNm. The global maximum of 90.76 kNm occurred in the upper grid, in member 291 at node 229. The mean value for the upper grid was 29.33 kNm, compared with 5.57 kNm for the lower subsystem and 3.18 kNm for the diagonal members; the corresponding maxima were 90.76, 16.04, and 5.80 kNm. Of the 405 members, 46 (11.4%) reached *M_r_* ≥ 50 kNm, whereas none reached 100 kNm. Compared with V2, V3 showed a higher overall mean but a lower maximum and a smaller standard deviation, indicating less pronounced variation in Mr across its members.

The reference Ammann A4 configuration exhibited a lower overall *M_r_* level than variants V1–V3, with a mean of 18.30 kNm, a median of 13.52 kNm, and a maximum of 74.50 kNm. Of its 363 members, 14 (3.9%) reached *M_r_* ≥ 50 kNm, whereas none reached 100 kNm. In contrast to the three new variants, Ammann A4 showed higher mean and maximum *M_r_* values in the lower subsystem and diagonals: their mean values were 9.48 and 4.86 kNm, with maxima of 42.86 and 21.04 kNm, respectively, while the upper-grid mean was 20.82 kNm. The Unibat reference configuration produced substantially smaller values, with a mean of 2.53 kNm, a median of 0.87 kNm, and a maximum of 12.10 kNm; none of its 321 members reached 50 kNm. Its mean values were 4.95 kNm in the upper grid, 0.92 kNm in the lower subsystem, and 0.42 kNm in the diagonal members. Accordingly, Unibat exhibited the lowest *M_r_* response among all five configurations, whereas the Ammann A4 results were closer to those of V1–V3, with a less pronounced difference between the upper-grid and lower-subsystem mean values.

### 4.3. Vertical Nodal Displacements (U_z_)

Vertical nodal displacements are presented in Figure 6 using a common colour scale based on their absolute magnitude, |*U_z_*|. In the numerical results, the original sign convention is retained: negative *U_z_* values denote downward movement along the global *Z* axis, and positive values denote upward movement. Unlike the member-level *F_x_* and *M_r_* results, each displacement value is assigned directly to a node and therefore requires no endpoint aggregation.

The statistical comparison includes all nodes in each configuration. The upper and lower nodal layers are examined separately, considering maximum downward displacement, signed mean, mean magnitude, median, and dispersion. Nodes belonging to the lower subsystem include its elevated end nodes. Zero values are retained in all relevant statistics and are not used to identify support conditions. The resulting means describe the sampled nodal response and depend on the distribution of nodes in each configuration.

- Variant V1 comprised 232 nodes, of which 161 exhibited downward displacement and 71 had a recorded *U_z_* value of zero. Across all nodes, *U_z_* ranged from −30.70 to 0.00 cm, with a mean of −8.57 cm, a median of −9.40 cm, and a standard deviation of 7.46 cm. The greatest downward displacement (*U_z_* = −30.70 cm) occurred at nodes 106, 169, and 195 in the upper nodal layer. The 208 upper-layer nodes had a mean displacement of −9.29 cm, compared with −2.35 cm for the 24 lower-layer nodes, with zero values included in both groups. Moreover, 16 (6.9%) nodes reached |*U_z_*| ≥ 20 cm, including 8 (3.4%) with |*U_z_*| ≥ 30 cm. The results indicate that the largest downward displacements occurred in the upper layer; the maximum downward displacement in the lower subsystem was only 7.10 cm.
- Variant V2 comprised 287 nodes, of which 214 exhibited downward displacement and 73 had a recorded *U_z_* value of zero. Across all nodes, *U_z_* ranged from −24.70 to 0.00 cm, with a mean of −8.79 cm, a median of −6.70 cm, and a standard deviation of 7.85 cm. The greatest downward displacement (*U_z_* = −24.70 cm) occurred at node 82 in the upper nodal layer. The 268 upper-layer nodes had a mean displacement of −9.12 cm, compared with −4.16 cm for the 19 lower-layer nodes, with zero values included in both groups. Overall, 38 (13.2%) nodes reached |*U_z_*| ≥ 20 cm, whereas none reached 30 cm. Compared with V1, V2 exhibited a smaller maximum downward displacement but a slightly greater mean displacement magnitude across all nodes (8.79 versus 8.57 cm). Its lower-layer maximum downward displacement was 18.80 cm.
- Variant V3 comprised 290 nodes, of which 202 exhibited downward displacement and 88 had a recorded *U_z_* value of zero. Across all nodes, *U_z_* ranged from −18.60 to 0.00 cm, with a mean of −6.30 cm, a median of −5.20 cm, and a standard deviation of 5.93 cm. The greatest downward displacement (*U_z_* = −18.60 cm) occurred at node 196 in the upper nodal layer. The 266 upper-layer nodes had a mean displacement of −6.65 cm, compared with −2.31 cm for the 24 lower-layer nodes, with zero values included in both groups. Overall, 84 (29.0%) nodes reached |*U_z_*| ≥ 10 cm, whereas none reached 20 cm. Compared with V1 and V2, V3 exhibited both the smallest maximum downward displacement and the lowest mean displacement magnitude across all nodes.

The reference Ammann A4 configuration comprised 263 nodes. Across all nodes, *U_z_* ranged from −32.20 to +1.80 cm, with a mean of −6.21 cm, a mean magnitude of 6.28 cm, a median of −2.40 cm, and a standard deviation of 8.42 cm. In total, 167 nodes moved downward, 8 exhibited upward displacement, and 88 had a recorded *U_z_* value of zero. The greatest downward displacement occurred at node 212 in the upper nodal layer. The 231 upper- and 32 lower-layer nodes had mean displacements of −6.76 and −2.21 cm, respectively. Thus, Ammann A4 developed a greater maximum downward displacement than V1, V2, or V3, despite its slightly lower mean displacement magnitude across all nodes than V3. By contrast, the Unibat reference configuration exhibited *U_z_* values ranging only from −3.20 to 0.00 cm. Across its 113 nodes, the mean displacement was −1.18 cm, the median was −1.10 cm, and the standard deviation was 1.03 cm; 81 nodes moved downward, and 32 had recorded zero values. The corresponding means for its 81 upper- and 32 lower-layer nodes were −1.09 and −1.41 cm. The greatest downward displacement occurred at node 46 in the upper layer, and no node reached |*U_z_*| ≥ 5 cm. Unibat therefore exhibited the smallest vertical displacement response among all five configurations.

## 5. Discussion

The combined results reveal clear trade-offs among the three variants in which the Ammann lines form the lower subsystem, rather than a single and consistent ordering of structural performance. Under the common loading, material, and member-section assumptions, V1 exhibited the smallest absolute axial-force and *M_r_* extremes among variants V1–V3 but also the greatest vertical displacement. V2 developed the largest local concentrations of both (*F_x_*) and (*M_r_*), despite exhibiting an intermediate displacement response. V3 produced the smallest vertical displacement and a lower *M_r_* maximum than V2, although its extreme axial force remained greater than that of V1. The reference configurations further demonstrate that rankings depend on the selected response criterion: Unibat showed the smallest *M_r_* values and vertical-displacement magnitudes, whereas Ammann A4 produced responses generally closer to those of the three new variants. Consequently, the observed differences cannot be attributed to relative heterogeneity alone, but must be interpreted in relation to topology, connectivity, and support arrangement.

### 5.1. Topological and Geometric Comparison of the Analysed Configurations

Variants V1–V3 share the same generative principle but represent distinct topological organisations. In each variant, the upper grid was derived from the non-duplicated edges of geometrically transformed Ammann A- and B-tiles, while selected continuous Ammann lines defined the lower tensile subsystem. The two subsystems were connected by diagonal members positioned according to the common geometric procedure described in Section 2.2. Because the tile arrangements were produced using repeated isometric transformations, the resulting configurations are periodic and should not be interpreted as direct reproductions of the original aperiodic Ammann A4 tessellation. Within this common framework, V1 represents the most continuous and redundant organisation, V2 exhibits a more directional arrangement of structural paths, and V3 contains a more selective distribution of connections between the upper and lower subsystems.

The differences between variants V1–V3 are partly described by relative heterogeneity. Relative heterogeneity is defined as the ratio of the number of non-A- and non-B-type tiles to the total tile count. The value is 1.5% for V1, in which almost the entire structural field is filled by A- and B-type tiles, except for the central square region. It increases to 53.9% for V2 and 56.7% for V3. These values distinguish the nearly complete tile-based arrangement of V1 from the more heterogeneous patterns of V2 and V3. However, relative heterogeneity is a descriptive geometric measure rather than an independent structural variable. It changes together with connectivity, member distribution, path continuity, and support arrangement and therefore cannot, by itself, explain differences in structural response. For the Ammann A4 reference configuration, relative heterogeneity is 0% because its upper grid follows the original A- and B-tile geometry. The descriptor is not applicable to Unibat, which is based on a square modular grid rather than Ammann tiles.

The topological descriptors reveal a substantial increase in discretisation from V1 to V2 and V3. V1 comprises 232 nodes and 328 members, whereas V2 contains 287 nodes and 404 members and V3 contains 290 nodes and 405 members. Thus, V2 and V3 have approximately 23–25% more nodes and members than V1. Nevertheless, their total member lengths remain relatively close: 1517.04 m for V1, 1488.45 m for V2, and 1567.52 m for V3. These lengths were calculated from the exported nodal coordinates and member connectivity. The principal difference therefore concerns the allocation of member length among the subsystems rather than its total value. Relative to V1, the upper-grid member length increases by approximately 15% in V2 and 17% in V3. By contrast, the lower-subsystem length decreases by approximately 27% in V2 and 14% in V3, while the total diagonal length decreases by approximately 31% and 23%, respectively. Consequently, configurations with similar total member lengths may differ considerably in member density, average member length, and the balance between their upper, lower, and diagonal systems.

The Ammann A4 reference configuration retains the original aperiodic tile organisation in its upper grid and follows the associated Ammann-line network in the lower subsystem [2]. Lower members that did not participate in connections with diagonal members were omitted. The resulting configuration comprises 263 nodes and 363 members, placing it between V1 and variants V2–V3 in terms of node and member counts. Its total member length is 1679.34 m, approximately 11.2% greater than that of V1. This difference is concentrated primarily in the lower subsystem, whose total length reaches 526.30 m, compared with 409.28 m in V1. In contrast, the total diagonal lengths of Ammann A4 and V1 are almost identical, amounting to 189.19 and 188.24 m, respectively. Ammann A4 also differs from V1–V3 in its irregular perimeter, which follows the geometry of the aperiodic tessellation and includes local outward projections. The Unibat reference configuration represents a fundamentally different organisational principle based on a regular modular space-frame system [23]. It contains only 113 nodes, about 51% fewer than V1, but has 321 members and therefore only about 2% fewer members. This apparent contrast results from a different member distribution: Unibat contains 144 upper-grid members, 49 lower members, and 128 diagonals, compared with 272, 24, and 32, respectively, in V1. Its total member length of 1979.77 m is approximately 22–28% greater than those of variants V1–V3 and 13.3% greater than that of Ammann A4. The diagonal system makes a major contribution, with a total length of 736.00 m. Unibat also has the longest average upper-grid members, at 5.83 m, whereas its lower members, averaging 8.25 m, are shorter on average than those of the Ammann-inspired configurations. These characteristics reflect its regular pyramidal topology rather than the line-based organisation adopted in the Ammann-inspired configurations.

The configurations also differ in their support arrangements, which follow the boundary rules adopted for each topology and are not geometrically identical. The geometric descriptors reported in Table 1 must therefore be interpreted as an interdependent set rather than as isolated explanatory variables. The comparison does not constitute a controlled test of relative heterogeneity alone, because node density, connectivity, member lengths, perimeter geometry, and support arrangement change simultaneously.

**Figure 7:**
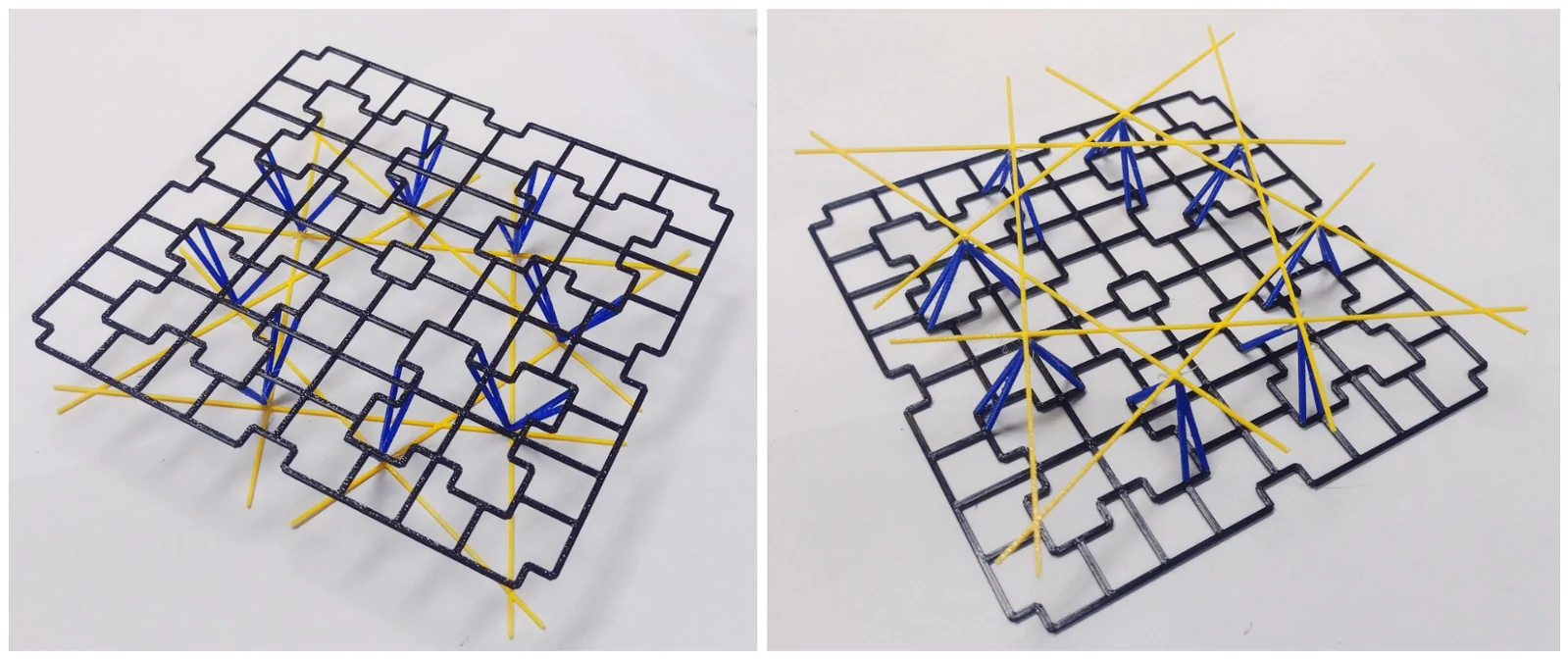
3D-printed physical mock-up of variant V1, shown in regular and inverted orientations for display and fabrication purposes. The black elements represent the tile-derived layer, the yellow rods indicate the Ammann-line-based tensile-oriented subsystem, and the blue members show the diagonal interlayer connectors. The inversion preserves the geometric relationships between the layers but does not represent a load-bearing test configuration.

The 3D-printed model of V1 shown in Figure 7 provides a physical representation of the proposed geometric construction. It illustrates the relationships among the upper tile-derived grid (printed in black), the continuous lower subsystem based on Ammann lines (printed in yellow), and the diagonal connectors (printed in blue). The prototype was used only to examine geometric relationships, spatial legibility, and physical assembly at model scale; it was not subjected to loading and does not provide experimental validation of the numerical structural results.

### 5.2. Structural Response of the Analysed Configurations to Vertical Loading

A structural comparison was conducted based on the calculation results presented in Section 4. The discussion focuses on axial forces *F_x_*, total resultant moment magnitudes *M_r_*, and vertical nodal displacements *U_z_*, which are detailed in Figure 5 and Figure 6 and Table 2, Table 3 and Table 4.

#### 5.2.1. Axial Force (*F_x_*)

The evaluation of axial-force distributions confirms that the lower subsystem in variants V1–V3 fulfills its intended design function by operating entirely under tension, although this qualitative classification is strictly based on the sign of the longitudinal forces rather than a comparison with bending verification. Specifically, all lower members in these variants exhibited tensile forces, as indicated by their negative *F_x_* values. The same tension-dominated response occurred in all 30 lower members of Ammann A4 and in 48 of the 49 lower members of Unibat. By contrast, the upper grids exhibited mixed axial action and therefore cannot be interpreted as purely compressive subsystems. The diagonal members were predominantly under compression in the Ammann-inspired configurations: all 32 diagonals were compressed in V1, while 22 of the 24 diagonals were compressed in V2, 20 of the 24 in V3, and 34 of the 36 in Ammann A4.

In both configurations V2 and V3, diagonal members subject to tension appear in the central part of the structure. In V3, these members are arranged in four symmetrical groups around the central zone defined by the layout of the bottom layer. In V2, two diagonals exhibited tensile axial forces in the current results. This tensile behavior stems from a local reversal of the force flow caused by the specific support conditions of all analysed structures, as detailed in Section 3. The top-layer supports restrained vertical translation (*U_z_*) while allowing horizontal movements and rotations. However, two supports situated on opposite edges featured additional constraints: one node was restrained in the *Y*-direction (*U_y_*), while the other was restrained in both the *X*- and *Y*-directions (*U_x_*, *U_y_*). Consequently, the system was no longer perfectly symmetrical in terms of stiffness. For configuration V3, an additional factor is the local, asymmetrical arrangement of diagonal members at the bottom-chord nodes, where three members converge at varying inclination angles relative to the *Z*-axis—a direct result of the top-layer layout. Consequently, within each of the four groups, one member is positioned distinctly asymmetrically relative to the other two, leading to an uneven force distribution.

In the Ammann-inspired configurations, the response combined direct load transfer through the upper grid to the upper supports with indirect transfer through predominantly compressive diagonals and the tensile lower subsystem to the lower supports. This interpretation describes the dominant axial-force pattern rather than a complete force-flow decomposition.

Although the intended functional separation was observed in all three new variants, the uniformity and concentration of axial forces differed substantially. V1 exhibited the smallest absolute axial-force extreme among V1–V3, at 324.68 kN, and the most uniform tension in the lower subsystem, whose coefficient of variation was only 4.0%. Its lower members therefore participated in the tensile response to a similar degree. V2 developed the largest absolute axial-force extreme, 448.32 kN, while its lower-subsystem CV increased to 19.2%. At the same time, its overall mean |*F_x_*|, 26.87 kN, was lower than the corresponding values for V1 and V3, at 37.10 and 32.27 kN. The combination of a relatively low global mean and the highest local extreme indicates that V2’s axial response was more strongly concentrated in selected members. V3 showed an intermediate maximum of 412.46 kN and a lower-subsystem CV of 24.0%. Its response was therefore less uniform than that of V1 but did not reproduce the particularly high local extreme found in V2.

Ammann A4 exhibited a maximum absolute axial force of 417.76 kN and a lower-subsystem CV of 38.6%, indicating a less uniform tensile distribution than in variants V1–V3. Unibat displayed a different response. Its absolute axial-force extreme, 186.87 kN, was the smallest among all five configurations, but its overall mean |*F_x_*|, 72.94 kN, was substantially greater than that of the Ammann-inspired configurations. Its diagonal system was also almost evenly split between tension and compression, with 66 tensile and 62 compressive members. This combination indicates broad mobilisation of axial action throughout the regular space-frame system, rather than concentration of high forces in a limited number of members. The result must nevertheless be considered alongside the much greater number and total length of diagonal members in Unibat.

#### 5.2.2. Total Resultant Moment Magnitude (*M_r_*)

The *M_r_* results show a different relationship among variants V1–V3. Their overall means ranged from 24.33 to 26.37 kNm: V2 had the lowest mean, V1 an intermediate value of 25.62 kNm, and V3 the highest mean. Their local maxima differed considerably: V1 had the lowest maximum, at 72.57 kNm; V3 reached 90.76 kNm; and V2 developed the highest value, at 129.12 kNm. V2 therefore combined the largest axial-force extreme with the largest local *M_r_* value, despite having the lowest mean *M_r_* among the three variants. In all three variants, the highest *M_r_* values occurred in the upper grid. The upper-grid means ranged from 26.74 to 30.06 kNm, whereas the lower-subsystem means remained between 5.55 and 5.82 kNm, and the diagonal means between 2.76 and 3.77 kNm. In conjunction with the axial-force patterns, these differences are consistent with a predominantly axial role for the lower and diagonal systems and a greater moment response in the upper grid.

The reference configurations show that low *M_r_* values can accompany different axial-force distributions. In Ammann A4, the overall mean *M_r_* was 18.30 kNm, and the maximum was 74.50 kNm. However, its lower subsystem and diagonals had higher *M_r_* maxima than those of V1–V3, reaching 42.86 and 21.04 kNm, respectively. This indicates a less pronounced separation of the reported moment response among the member groups. Unibat produced the lowest *M_r_* response, with a mean of 2.53 kNm and a maximum of 12.10 kNm. Together with its high mean axial-force magnitude, this pattern is consistent with a response dominated more strongly by axial action. It should not, however, be interpreted as evidence of greater material efficiency because Unibat has the greatest total member length and a substantially more extensive diagonal system.

#### 5.2.3. Vertical Nodal Displacements (*U_z_*)

The displacement results demonstrate that uniform axial-force distribution does not necessarily correspond to smaller maximum vertical displacement. V1, despite its uniform lower-layer tension and comparatively low axial-force and *M_r_* extremes, exhibited the greatest maximum displacement among the three new variants. Its maximum downward displacement was 30.70 cm, and the mean displacement magnitude across all nodes was 8.57 cm. V2 reduced the maximum to 24.70 cm, but its all-node mean magnitude was slightly greater, at 8.79 cm. V3 produced the smallest response on both measures, with a maximum of 18.60 cm and an all-node mean magnitude of 6.30 cm. V3 therefore showed the lowest vertical deformation among the three new variants under the adopted assumptions, although this advantage came with greater axial-force extremes than in V1. The distinction between the maximum and the nodal mean is important: the latter depends on the distribution of sampled nodes, including nodes with zero displacement. The comparison again shows that no variant is preferable across all response criteria.

Ammann A4 developed a maximum downward displacement of 32.20 cm, exceeding that of V1, but its mean displacement magnitude across all nodes was 6.28 cm, close to that of V3. Eight nodes exhibited small upward displacements, with a maximum upward displacement of +1.80 cm, demonstrating local reversal within a non-uniform deformation field. Unibat showed a markedly smaller displacement response: its maximum downward displacement was 3.20 cm, and its all-node mean magnitude was 1.18 cm. This behaviour is consistent with its regular pyramidal organisation and extensive diagonal connectivity. However, this displacement advantage comes with a total member length of 1979.77 m, compared with 1488.45–1567.52 m for variants V1–V3, and a different support arrangement. The comparison therefore identifies a trade-off between low deformation, structural regularity, and the geometric extent of the member system.

The differing responses of V2 and V3 are particularly important because these variants have similar numbers of nodes and members and comparable relative heterogeneity values. Nevertheless, V2 developed higher local *F_x_* and *M_r_* maxima and greater maximum and mean vertical displacement magnitudes than V3. This finding shows that relative heterogeneity alone does not capture the structural consequences of connectivity, directional continuity, and support placement. Conversely, V1 shows that a highly continuous configuration with uniform lower-layer tension may remain comparatively flexible. The structural response is therefore governed by the combined organisation of the upper grid, tensile paths, diagonal connections, and supports rather than by tile coverage or member count considered separately.

Within the scope of the adopted numerical models, using Ammann lines as the organising geometry of the lower subsystem successfully generated continuous tensile paths in variants V1–V3. However, the results do not establish code compliance or determine the load-bearing capacity of the configurations. They are limited to the adopted member model, common sections and materials, geometry-dependent support arrangements, and the KOMB1 loading condition. Connection design, member optimisation, alternative loading patterns, and experimental loading of a physical model remain outside the present study. Accordingly, the comparison provides evidence of relative structural response and load-transfer organisation, not a final ranking of buildable structural designs.

### 5.3. General Observations

The distinct structural behaviors, shifting performance rankings, and deformation profiles among variants V1, V2, and V3 are not merely descriptive data variations. Instead, they are inherently rooted in the underlying mechanics of force flow organization. The transition from the highly uniform force distribution in V1 to the localized stress concentrations in V2 and V3, alongside the behavior of the reference configurations, is governed by the global interplay between the continuity of the upper chord grid, the physical proportions of the lower chord paths, and the spatial, boundary-induced distribution of the diagonal members (oblique rods).

#### 5.3.1. Force Flow Diffusion via Upper Chord Continuity and Homogeneity (V1)

The highly uniform distribution of lower-subsystem axial forces observed in V1 is a direct consequence of its topological arrangement, characterized by an exceptionally low relative heterogeneity of 1.5%. In this configuration, uniform A- and B-type tiles fill almost the entire structural field, allowing the upper subsystem to act as a highly continuous, redundant structural mattress. When external vertical loads are applied, this uninterrupted grid effectively diffuses stress concentrations globally before they can reach the lower subsystem. Furthermore, V1 allocates a significantly higher proportion of total member length to the lower subsystem and diagonals compared to V2 and V3. This extended, redundant network ensures that the internal force flow is distributed across a wider array of structural paths in small, incremental steps.

This mechanism is quantitatively proven by the exceptionally low coefficient of variation (CV = 4.0%) of tensile forces in the lower subsystem of V1 and its low absolute axial-force extreme (324.68 kN), demonstrating that all lower members participate in the tensile response to a nearly equal degree. Additionally, the continuity of the upper chord grid successfully homogenizes the bending demand, resulting in the lowest maximum moment of 72.57 kNm. However, while this extensive load-sharing mechanism minimizes local peak forces, the lower nodal density reduces local spatial stiffness. This makes the continuous configuration comparatively flexible, which directly accounts for the greatest vertical displacement (30.70 cm) recorded among the three new variants. This mechanism is further validated by the Ammann A4 reference configuration; its even longer lower subsystem (526.30 m) provides an extensively distributed tensile network that similarly diffuses forces, though its inherent aperiodicity yields a higher variation (CV = 38.6%) and a maximum displacement of 32.20 cm.

#### 5.3.2. Path Proportions and Force Bottlenecks in V2 and V3

In contrast, the severe local concentrations of both axial forces *F_x_* and bending moments *M_r_* in V2 and V3 are a direct consequence of a significantly altered lower chord path proportion and increased system heterogeneity. In V2, the total length of the lower subsystem decreases by approximately 27% relative to V1, meaning the tensile lower network is stripped of alternative paths and condensed into highly directional, specific structural routes. According to the principles of structural mechanics, force flow naturally scales with relative stiffness, seeking the shortest and most rigid path to the supports. The shortened lower chord paths in V2 act as high-stiffness structural highways that aggressively attract a disproportionate volume of the global force flow.

Because the relative heterogeneity jumps sharply to 53.9% in V2, these rigid paths intersect with geometric irregularities, creating severe structural bottlenecks. The massive accumulation of force flow at these specific topological intersections manifests as the highest local axial-force extreme (448.32 kN) and a sharp increase in the lower-subsystem variation (CV = 19.2%), despite having the lowest overall mean force (26.87 kN). Furthermore, these severe axial force bottlenecks at the geometric discontinuities induce secondary structural actions and severe eccentricities, triggering a massive surge in the localized moment response up to 129.12 kNm. Consequently, V2 simultaneously develops the largest axial-force extreme and the largest local *M_r_* value. For V3, the lower-subsystem CV rises even further to 24.0% with an intermediate extreme of 412.46 kN, proving that the force flow in both V2 and V3 shifts from a diffused global matrix to highly localized structural streams where the rigid lower paths intersect the heterogeneous grid.

#### 5.3.3. Web Member Mediation, Displacement Trade-Offs, and Boundary Conditions

The organization, deformation control, and potential disruption of the force flow between the upper and lower layers are structurally mediated by the diagonal web members, whose total lengths are reduced by 31% in V2 and 23% in V3 compared to V1. V1 has fewer nodes overall than V2 and V3, but more diagonal members; all 32 diagonals carry compressive axial forces in the current results. V2 and V3 each contain 24 diagonal members, compared with 32 in V1. Their mean diagonal lengths are 5.45 and 6.04 m, respectively, compared with 5.88 m in V1; thus, the diagonals are shorter on average in V2 but not in V3. Crucially, the force flow in V2 and V3 undergoes a local reversal in the central zone, where diagonal members exhibit tensile behavior. This phenomenon is a direct mechanical response to the asymmetric boundary conditions (the specific *U_x_* and *U_y_* restraints on opposite edges), which break the structural symmetry of stiffness. In V3, this effect is further amplified by the local, asymmetrical arrangement of diagonal members at the bottom-chord nodes, where three members converge at varying inclination angles. This asymmetric layout forces the diagonal members to act as sharp, direct vectors that dump forces abruptly and unevenly, leading to local force concentrations.

The structural consequences of this web distribution are clearly manifested in the vertical displacement responses, highlighting that relative heterogeneity alone (53.9% for V2 vs. 56.7% for V3) cannot capture the true nature of connectivity. Although V2 and V3 share similar node and member counts, V3 successfully optimizes the force flow vectors through its selective connectivity, enhancing the effective truss-action depth of the system. This optimization allows V3 to achieve the smallest maximum vertical displacement (18.60 cm) and the lowest mean magnitude (6.30 cm) among the three variants, effectively trading a higher local axial-force extreme for a substantial gain in global vertical stiffness.

This line-based mechanical behavior contrasts sharply with the Unibat reference configuration, which operates on a fundamentally different organizational principle. Unibat bypasses line-based force tracing by deploying a regular, modular space-frame system where the diagonal system makes a massive contribution (736.00 m, approximately 3.8 times that of V1, 5.5 times that of V2, and 5.0 times that of V3). This exceptionally dense, three-dimensional pyramidal network provides supreme global shear stiffness. Instead of tracing forces along individual linear paths, Unibat distributes loads uniformly across its highly rigid spatial cell matrix, resulting in a broad mobilization of axial action (almost evenly split between 66 tensile and 62 compressive diagonals), a comparatively low bending-moment response (overall mean *M_r_* of 2.53 kNm), and an ultra-low maximum downward displacement of 3.20 cm, albeit at the expense of a 26–33% greater total member length.

Ultimately, within the scope of the adopted numerical assumptions, using Ammann lines successfully generated continuous tensile paths across all variants. However, these findings identify clear architectural and structural trade-offs between low deformation, localized force concentrations, topological regularity, and the geometric extent of the member system, rather than establishing a single, universally preferable design configuration.

## 6. Conclusions and Future Outlook

### 6.1. Main Findings

This study demonstrated that selected geometric principles derived from the Ammann A4 tessellation can be translated into periodic two-layer structural configurations in which continuous Ammann lines organise the lower subsystem. The numerical results confirmed the tensile character of this layout under the analysed load combination: in variants V1–V3, all lower members operated under tensile forces, while the diagonal members worked predominantly under compression. Meanwhile, the upper grid accommodated mixed axial action and carried most of the total moment response.

No single variant was superior across all criteria. V1 provided the most uniform tensile-force distribution along with the smallest absolute axial-force and *M_r_* extremes among the three new variants, but it exhibited the greatest vertical displacement. V2 developed the strongest local concentrations of axial force and total moment, whereas V3 produced the smallest vertical displacement while retaining greater axial-force extremes than V1. Unibat reference configuration exhibited the smallest *M_r_* values and vertical-displacement magnitudes, but this response was associated with a more extensive diagonal system, greater total member length, and a different support arrangement. The results therefore show that relative heterogeneity, member count, or tile coverage cannot independently explain structural behavior. Connectivity, directional continuity, member distribution, and support placement must be considered collectively.

### 6.2. Directions for Future Research

The study offers a comparative evaluation rather than code-compliant structural designs. Future research should isolate individual geometric variables using parametric models with controlled support conditions, member density, and total material quantity. Additionally, subsequent efforts should focus on cross-section optimization, member stability and buckling assessments, connection design, alternative and asymmetric loading conditions, and non-linear analysis. Consideration should also be given to replacing the bottom-layer members with pre-tensioned cables. Finally, load-bearing physical prototypes should be tested to verify the numerical load-transfer mechanisms and to evaluate the practical fabrication and assembly of Ammann-inspired structural systems.

## Figures and Tables

**Table 1:** Geometric and topological descriptors of the analysed configurations. Bold rows indicate aggregate values, while the rows immediately below provide a breakdown into their constituent components.

Descriptor	Var. 1	Var. 2	Var. 3	Ammann	Unibat
Relative heterogeneity [%]	1.5	53.9	56.7	0	n/a
**Total number of nodes**	232	287	290	263	113
Number of upper-layer nodes	208	268	266	231	81
Number of lower-layer nodes	24	19	24	32	32
**Total number of members**	328	404	405	363	321
Number of upper-layer members	272	360	357	297	144
Number of lower-layer members	24	20	24	30	49
Number of diagonal members	32	24	24	36	128
**Total number of supports**	71	73	86	87	32
Number of fully pinned supports (*U_x_*, *U_y_*, *U_z_*)	17	13	17	24	1
Number of partially constrained supports (*U_y_*, *U_z_*)	1	1	1	1	1
Number of vertical supports (*U_z_*)	53	59	68	62	30
Number of upper-layer supports	55	61	70	64	32
Number of lower-layer supports	16	12	16	23	0
**Total member length** [m]	1517.04	1488.45	1567.52	1679.34	1979.77
Total upper-layer member length [m]	919.52	1059.53	1072.36	963.86	839.52
Total lower-layer member length [m]	409.28	298.12	350.20	526.30	404.25
Total diagonal member length [m]	188.24	130.80	144.96	189.19	736.00
**Average member length (all members)** [m]	4.625	3.684	3.870	4.626	6.168
Average upper-layer member length [m]	3.381	2.943	3.004	3.245	5.830
Average lower-layer member length [m]	17.053	14.906	14.592	17.543	8.250
Average diagonal member length [m]	5.883	5.450	6.040	5.255	5.750
**Total external live load on the upper layer [kN]**	1064.05	1063.59	1061.20	1037.05	997.80
**Total mass of members** [kg]	64 948	70 122	71 972	69 873	67 143
Mass of members in the upper layer [kg]	54 084	62 319	63 074	56 692	47 346
Mass of members in the lower layer [kg]	7 553	5 502	6 298	9 853	7 143
Mass of diagonal members [kg]	3 311	2 301	2 600	3 328	12 354

**Table 2:** Summary of member axial-force response for the five structural configurations.

Configuration	Members	Overall *F_x_* Range (kN)	Mean |*F_x_*| (kN)	Lower-Subsystem *F_x_* Range (kN)	Lower Mean *F_x_* (kN)	Lower CV (%)	Diagonal T/C (*n*)
V1	328	−324.68 to +71.98	37.10	−324.68 to −291.79	−308.58	4.0	0/32
V2	404	−448.32 to +133.01	26.87	−448.32 to −243.54	−350.92	19.2	2/22
V3	405	−412.46 to +144.74	32.27	−412.46 to −193.84	−292.04	24.0	4/20
Ammann A4	363	−417.76 to +114.16	29.86	−417.76 to −90.55	−246.73	38.6	2/34
Unibat	321	−186.87 to +184.31	72.94	−186.87 to +8.88	−116.53	40.7	66/62

Note. For each member, we used the signed endpoint value with the greatest absolute magnitude. Negative *F_x_* values denote tension and positive values denote compression. T/C gives the number of diagonal members in tension and compression, respectively. Lower CV (%) = 100 × SD(|*F_x_*|)/mean(|*F_x_*|), using the population standard deviation of member force magnitudes. Each member has equal weight. The statistics are comparative response descriptors rather than direct measures of structural efficiency.

**Table 3:** Summary of member *M_r_* response for the five structural configurations.

Configuration	Members	Overall *M_r_* Range (kNm)	Mean |*M_r_*| (kNm)	Lower-Subsystem *M_r_* Range (kNm)	Lower Mean *M_r_* (kNm)	Lower CV (%)	Diagonal (*M_r_*) Range (kNm)
V1	328	1.33 to 72.57	25.62	2.15 to 7.76	5.82	43.7	1.33 to 3.92
V2	404	1.35 to 129.12	24.33	2.28 to 13.84	5.55	74.8	1.35 to 4.52
V3	405	0.49 to 90.76	26.37	0.49 to 16.04	5.57	86.9	1.22 to 5.80
Ammann A4	363	0.53 to 74.50	18.30	0.63 to 42.86	9.48	94.2	0.53 to 21.04
Unibat	321	0.21 to 12.10	2.53	0.80 to 1.04	0.92	5.8	0.29 to 0.75

Note: Each member is represented by the maximum *M_r_* value recorded at either endpoint. Lower CV (%) = 100 × SD(*M_r_*)/mean(*M_r_*), using the population standard deviation. Each member has equal weight. Since *M_r_* is non-negative, absolute-value notation and tension/compression classification are not used.

**Table 4:** Summary of vertical nodal displacement *U_z_* response for the five structural configurations.

Configuration	Total Nodes (*n*)	Overall *U_z_* Range (cm)	All-Node Mean |*U_z_*| (cm)	Lower-Layer *U_z_* Range (cm)	Lower Mean |*U_z_*| (cm)	Lower CV (%)	Upper-Layer *U_z_* Range (cm)
V1	232	−30.70 to 0.00	8.57	−7.10 to 0.00	2.35	141.4	−30.70 to 0.00
V2	287	−24.70 to 0.00	8.79	−18.80 to 0.00	4.16	158.4	−24.70 to 0.00
V3	290	−18.60 to 0.00	6.30	−10.70 to 0.00	2.31	158.0	−18.60 to 0.00
Ammann A4	263	−32.20 to +1.80	6.28	−28.50 to +1.60	2.31	244.4	−32.20 to +1.80
Unibat	113	−3.20 to 0.00	1.18	−3.10 to −0.20	1.41	65.2	−3.20 to 0.00

Note: All statistics include all nodes in the relevant configuration or nodal layer, including recorded zero values. No free-node filtering is applied, and zero displacement is not used to identify supports. Each node has equal weight. Lower CV (%) = 100 × SD(|*U_z_*|)/mean(|*U_z_*|), using the population standard deviation of displacement magnitudes. Negative *U_z_* values denote downward displacement and positive values denote upward displacement. Lower-layer nodes include the elevated end nodes of the lower subsystem.

## Data Availability

The original contributions presented in this study are included in the article and Supplementary Materials. Further inquiries regarding the raw data supporting the conclusions of this article can be directed to the corresponding author.

## Supplementary Materials

The following supporting information can be downloaded at: https://www.mdpi.com/article/10.3390/ma19214461/s1, Model S1: Structural variant V1—interactive 3D model (OBJ format); Model S2: Structural variant V2—interactive 3D model (OBJ format); Model S3: Structural variant V3—interactive 3D model (OBJ format); Model S4: Structural variant Ammann—interactive 3D model (OBJ format); Model S5: Structural variant Unibat—interactive 3D model (OBJ format); Figure S1: Structural responses of variant Ammann: axial forces (*F_x_*) (top), bending moments (*M_y_*) (middle) and vertical nodal displacements (*U_z_*) (bottom); Figure S2: Structural responses of variant Unibat: axial forces (*F_x_*) (top), bending moments (*M_y_*) (middle) and vertical nodal displacements (*U_z_*) (bottom); Figure S3: Torsional moment (*M_x_*) plots for the different structural configurations: V1 (top left), V2 (middle left), V3 (bottom left), Ammann A4 (top right), and Unibat (middle right); Table S1: Detailed descriptive statistics of member axial forces (*F_x_*) by member group; Table S2: Detailed descriptive statistics of resultant moments (*M_r_*) by member group for all five structural configurations; Table S3: Detailed descriptive statistics of resultant moments normalised by member length (*M_rn_*) by member group; Table S4: Detailed descriptive statistics of member torsional moments (*M_x_*) by member group; Table S5: Descriptive statistics of nodal vertical displacements (*U_z_*) for the five structural configurations, reported for all nodes and separately for the upper and lower layers.
