# Supplementary material for: Comparative Structural Behaviour of Periodic Two-Layer Spatial Grids Inspired by Ammann A4 Geometry
Source: Materials (Basel). 2026 Oct 23;19(21):4461. doi: 10.3390/ma19214461 (PMC13764467; doi:10.3390/ma19214461)
Supplement: Supplementary file 1 [file materials-19-04461-s001.zip › Figure S2.pdf]

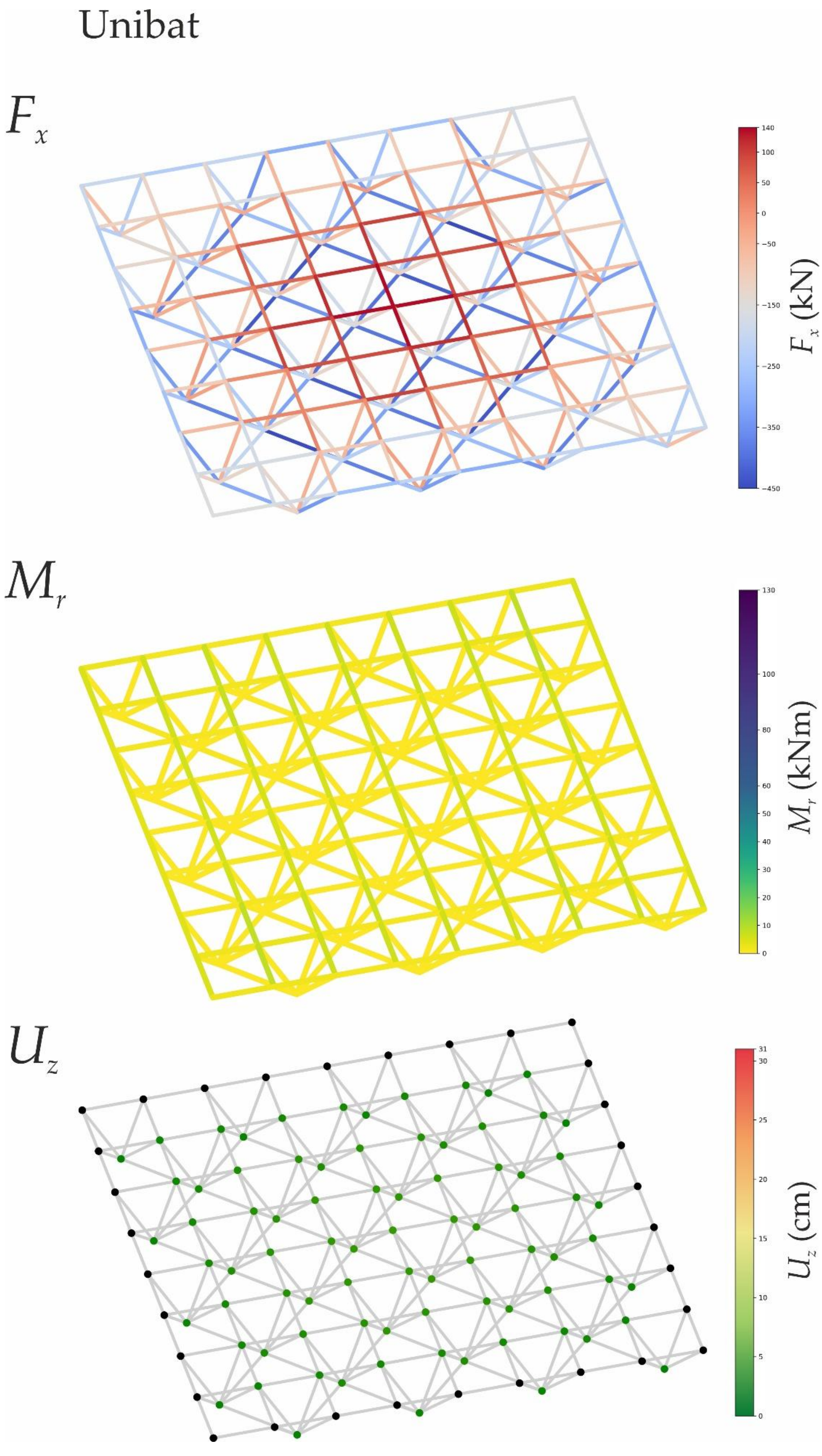

Figure S2. Structural responses of variant Unibat: axial forces (F_x) (top), bending moments (M_y) (middle) and vertical nodal displacements (U_z) (bottom).
