# Supplementary material for: Comparative Structural Behaviour of Periodic Two-Layer Spatial Grids Inspired by Ammann A4 Geometry
Source: Materials (Basel). 2026 Oct 23;19(21):4461. doi: 10.3390/ma19214461 (PMC13764467; doi:10.3390/ma19214461)
Supplement: Supplementary file 1 [file materials-19-04461-s001.zip › Figure S3.pdf]

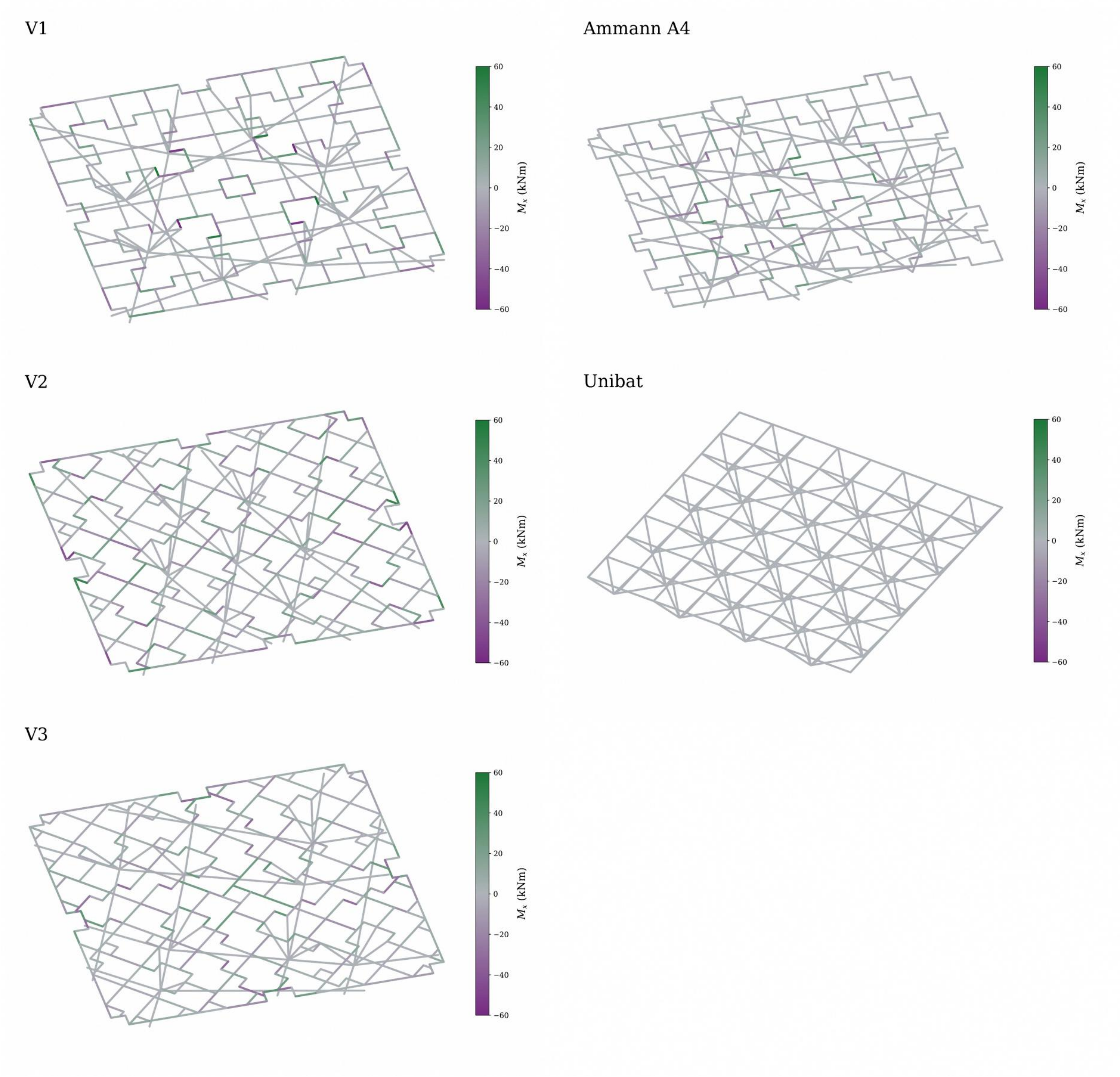

Figure S3. Torsional moment (Mx) plots for the different structural configurations: V1 (top left), V2 (middle left), V3 (bottom left), Ammann A4 (top right), and Unibat (middle right).
