# Supplementary material for: Comparative Structural Behaviour of Periodic Two-Layer Spatial Grids Inspired by Ammann A4 Geometry
Source: Materials (Basel). 2026 Oct 23;19(21):4461. doi: 10.3390/ma19214461 (PMC13764467; doi:10.3390/ma19214461)
Supplement: Supplementary file 1 [file materials-19-04461-s001.zip › Table_S1.pdf]

Table S1. Detailed descriptive statistics of member axial forces F_x by member group.

Configuration	Member group	n	≥50 / ≥100	Minimum	Maximum	Mean	Median	SD	Mean $\|F_x\|$	CV* (%)
**V1**	All members	328	64/24	−324.68	71.98	−19.23	−1.73	84.87	37.1	—
V1	Upper grid	272	20/0	−40.03	61.69	−1.47	−1.77	20.26	12.01	—
V1	Lower subsystem	24	24/24	−324.68	−291.79	−308.58	−310.98	12.21	308.58	4
V1	Diagonals	32	20/0	5.08	71.98	46.79	53.48	19.85	46.79	—
**V2**	All members	404	37/24	−448.32	133.01	−18.51	−1.48	80	26.87	—
V2	Upper grid	360	8/0	−73.10	14.99	−4.51	−1.48	12.67	7.38	—
V2	Lower subsystem	20	20/20	−448.32	−243.54	−350.92	−343.99	67.31	350.92	19.2
V2	Diagonals	24	9.kwi	−3.25	133.01	48.58	37.78	45.11	49.06	—
**V3**	All members	405	56/28	−412.46	144.74	−10.52	2.92	76.53	32.27	—
V3	Upper grid	357	16/0	−64.59	75.33	3.86	3.14	17.99	12.85	—
V3	Lower subsystem	24	24/24	−412.46	−193.84	−292.04	−289.46	70.09	292.04	24
V3	Diagonals	24	16.kwi	−19.13	144.74	57.15	56.65	46.81	61.48	—
**Ammann A4**	All members	363	44/31	−417.76	114.16	−15.27	0.17	76.85	29.86	—
Ammann A4	Upper grid	297	1 / 0	−34.48	51,87	1,28	0,17	10,78	6,57	—
Ammann A4	Lower subsystem	30	30 / 28	−417.76	−90.55	−246.73	−243.95	95,3	246,73	38,6
Ammann A4	Diagonals	36	3 / 13	−3.64	114,16	41,07	37,79	30,82	41,35	—
**Unibat**	All members	321	183 / 103	−186.87	184,31	7,07	−0.13	88,73	72,94	—
Unibat	Upper grid	144	80 / 56	−92.12	184,31	56,69	51,61	76,27	77,48	—
Unibat	Lower subsystem	49	43 / 35	−186.87	8,88	−116.53	−137.56	49,41	117,26	40,7
Unibat	Diagonals	128	60 / 12	−122.30	90,79	−1.43	−2.48	59,77	50,86	—

Force statistics are expressed in kN. The threshold column reports cumulative counts for $|F_x| \geq 50$ kN and $|F_x| \geq 100$ kN.

Note: Each member is represented by the end force with the greatest absolute magnitude, retaining its sign. SD is the population standard deviation of signed values. CV = 100 × SD($|F_x|$)/mean($|F_x|$), reported only for the lower subsystem and calculated before rounding.*
