# Supplementary material for: Comparative Structural Behaviour of Periodic Two-Layer Spatial Grids Inspired by Ammann A4 Geometry
Source: Materials (Basel). 2026 Oct 23;19(21):4461. doi: 10.3390/ma19214461 (PMC13764467; doi:10.3390/ma19214461)
Supplement: Supplementary file 1 [file materials-19-04461-s001.zip › Table_S2.pdf]

Table S2. Detailed descriptive statistics of resultant moments M_r by member group for all five structural configurations.

Configuration	Member group	n	$M_r \geq 50$ / ≥ 100 kNm (n)	Minimum M_r (kNm)	Maximum M_r (kNm)	Mean M_r (kNm)	Median M_r (kNm)	SD (kNm)	CV, lower subsystem (%)
**V1**	All members	328	58/0	1.33	72.57	25.62	21.4	19.77	—
V1	Upper grid	272	58/0	1.45	72.57	30.06	26.63	18.84	—
V1	Lower subsystem	24	0/0	2.15	7.76	5.82	7.54	2.54	43.7
V1	Diagonals	32	0/0	1.33	3.92	2.76	2.79	0.72	—
**V2**	All members	404	38/4	1.35	129.12	24.33	21.91	20.04	—
V2	Upper grid	360	38/4	1.45	129.12	26.74	23.86	19.9	—
V2	Lower subsystem	20	0/0	2.28	13.84	5.55	3.87	4.15	74.8
V2	Diagonals	24	0/0	1.35	4.52	3.77	4.15	0.98	—
**V3**	All members	405	46/0	0.49	90.76	26.37	24.01	18.58	—
V3	Upper grid	357	46/0	2.89	90.76	29.33	26.55	17.77	—
V3	Lower subsystem	24	0/0	0.49	16.04	5.57	3.35	4.84	86.9
V3	Diagonals	24	0/0	1.22	5.8	3.17	2.96	1.4	—
**Ammann A4**	All members	363	14/0	0.53	74.5	18.3	13.52	15.53	—
Ammann A4	Upper grid	297	14/0	0.97	74.5	20.82	16.58	15.75	—
Ammann A4	Lower subsystem	30	0/0	0.63	42.86	9.48	7.47	8.93	94.2
Ammann A4	Diagonals	36	0/0	0.53	21.04	4.86	3.05	4.59	—
**Unibat**	All members	321	0/0	0.21	12.1	2.53	0.87	3.31	—
Unibat	Upper grid	144	0/0	0.21	12.1	4.95	4.5	3.71	—
Unibat	Lower subsystem	49	0/0	0.8	1.04	0.92	0.9	0.05	5.8
Unibat	Diagonals	128	0/0	0.29	0.75	0.42	0.35	0.13	—

Note: Each member is represented by the maximum M_r value recorded at either of its two endpoints. Member groups are identified from the supplied geometry. The threshold column reports the numbers of members with $M_r \geq 50$ kNm and $M_r \geq 100$ kNm, respectively; these counts are cumulative. SD denotes the population standard deviation. CV is calculated as 100 × SD/mean using unrounded values and is reported only for the lower subsystem, following the original table format. All reported M_r values are non-negative
