# Supplementary material for: Comparative Structural Behaviour of Periodic Two-Layer Spatial Grids Inspired by Ammann A4 Geometry
Source: Materials (Basel). 2026 Oct 23;19(21):4461. doi: 10.3390/ma19214461 (PMC13764467; doi:10.3390/ma19214461)
Supplement: Supplementary file 1 [file materials-19-04461-s001.zip › Table_S3.pdf]

Table S3. Detailed descriptive statistics of resultant moments normalised by member length M_{rn} by member group.

Configuration	Member group	n	≥50 / ≥100	Minimum	Maximum	Mean	Median	SD	CV, lower subsystem (%)
**V1**	All members	328	0 / 0	0.21	36.28	8.86	7.8	8	—
V1	Upper grid	272	0 / 0	0.30	36.28	10.6	10.43	7.71	—
V1	Lower subsystem	24	0 / 0	0.21	0.4	0.32	0.35	0.08	24.3
V1	Diagonals	32	0 / 0	0.22	0.65	0.47	0.49	0.13	—
**V2**	All members	404	6 / 2	0.21	382.8	10.39	7.84	22.44	—
V2	Upper grid	360	6 / 2	0.27	382.8	11.6	8.54	23.49	—
V2	Lower subsystem	20	0 / 0	0.21	0.54	0.33	0.31	0.11	32.9
V2	Diagonals	24	0 / 0	0.25	0.83	0.69	0.77	0.18	—
**V3**	All members	405	8 / 1	0.05	277.23	11.33	7.72	17.21	—
V3	Upper grid	357	8 / 1	0.72	277.23	12.79	8.88	17.83	—
V3	Lower subsystem	24	0 / 0	0.05	0.59	0.35	0.24	0.17	47.8
V3	Diagonals	24	0 / 0	0.2	0.96	0.53	0.49	0.23	—
**Ammann A4**	All members	363	0 / 0	0.07	38.09	6.16	4.71	6.02	—
Ammann A4	Upper grid	297	0 / 0	0.23	38.09	7.36	5.98	6.02	—
Ammann A4	Lower subsystem	30	0 / 0	0.07	1.13	0.49	0.45	0.26	53.3
Ammann A4	Diagonals	36	0 / 0	0.09	4.76	1.01	0.63	1.05	—
**Unibat**	All members	321	0 / 0	0.04	2.16	0.45	0.12	0.6	—
Unibat	Upper grid	144	0 / 0	0.04	2.16	0.89	0.8	0.66	—
Unibat	Lower subsystem	49	0 / 0	0.10	0.13	0.11	0.11	0.01	6.1
Unibat	Diagonals	128	0 / 0	0.05	0.13	0.07	0.06	0.02	—

Moment-per-length statistics are expressed in kNm/m. The threshold column reports cumulative counts for $M_{rn} \geq 50$ kNm/m and $M_{rn} \geq 100$ kNm/m.

Note: $M_{rn} = M_r/L$. Each member is represented by the greater of its two reported end values. Statistics are calculated directly from the supplied normalised values, without additional length weighting. SD denotes the population standard deviation. CV = 100 × SD/mean, calculated before rounding. All values are non-negative.
