# Supplementary material for: Comparative Structural Behaviour of Periodic Two-Layer Spatial Grids Inspired by Ammann A4 Geometry
Source: Materials (Basel). 2026 Oct 23;19(21):4461. doi: 10.3390/ma19214461 (PMC13764467; doi:10.3390/ma19214461)
Supplement: Supplementary file 1 [file materials-19-04461-s001.zip › Table_S4.pdf]

Table S4. Detailed descriptive statistics of member torsional moments M_x by member group.

Configuration	Member group	n	≥50 / ≥100	Minimum	Maximum	Mean	Median	SD	Mean $\lvert M_x \rvert$	CV* (%)
**V1**	All members	328	8/0	−59.84	59,29	−0.01	0	14,93	9,48	—
V1	Upper grid	272	8/0	−59.84	59,29	−0.01	0,01	16,39	11,42	—
V1	Lower subsystem	24	0/0	−0.17	0,17	0	0	0,09	0,06	133,9
V1	Diagonals	32	0/0	−0.26	0,27	0	−0.01	0,12	0,09	—
**V2**	All members	404	0/0	−48.54	48,5	−0.04	0	14,14	9,86	—
V2	Upper grid	360	0/0	−48.54	48,5	−0.05	−0.19	14,98	11,05	—
V2	Lower subsystem	20	0/0	−0.41	0,38	−0.01	0	0,18	0,1	159,6
V2	Diagonals	24	0/0	−0.29	0,3	0	0,02	0,18	0,15	—
**V3**	All members	405	0/0	−30.80	31,69	0,1	0,01	11,32	7,97	—
V3	Upper grid	357	0/0	−30.80	31,69	0,11	0,75	12,05	9,01	—
V3	Lower subsystem	24	0/0	−0.06	1,01	0,12	0	0,33	0,13	243,3
V3	Diagonals	24	0/0	−0.82	0,73	−0.07	−0.15	0,46	0,38	—
**Ammann A4**	All members	363	0/0	−30.37	38,05	0,25	0	9,6	6,27	—
Ammann A4	Upper grid	297	0/0	−30.37	38,05	0,32	0,33	10,61	7,61	—
Ammann A4	Lower subsystem	30	0/0	−5.26	0,43	−0.21	0	0,96	0,27	350,3
Ammann A4	Diagonals	36	0/0	−1.12	1,11	0,02	0,04	0,32	0,21	—
**Unibat**	All members	321	0/0	−1.56	1,24	0	0	0,34	0,18	—
Unibat	Upper grid	144	0/0	−1.56	1,24	0	0	0,51	0,39	—
Unibat	Lower subsystem	49	0/0	−0.05	0,05	0	0	0,03	0,02	108
Unibat	Diagonals	128	0/0	−0.03	0,03	0	0	0,01	0,01	—

Moment statistics are expressed in kNm. The threshold column reports cumulative counts for $\lvert M_x \rvert \geq 50$ kNm and $\lvert M_x \rvert \geq 100$ kNm.

Note: Each member is represented by the end moment with the greatest absolute magnitude, retaining its sign. SD is the population standard deviation of signed values. CV = 100 × SD($\lvert M_x \rvert$)/mean($\lvert M_x \rvert$), reported only for the lower subsystem. Large CV values should be interpreted alongside the small absolute moment magnitudes. Displayed zeros may represent small non-zero values rounded to two decimal places.*
