# Supplementary material for: Comparative Structural Behaviour of Periodic Two-Layer Spatial Grids Inspired by Ammann A4 Geometry
Source: Materials (Basel). 2026 Oct 23;19(21):4461. doi: 10.3390/ma19214461 (PMC13764467; doi:10.3390/ma19214461)
Supplement: Supplementary file 1 [file materials-19-04461-s001.zip › Table_S5.pdf]

Table S5. Descriptive statistics of nodal vertical displacements U_z for the five structural configurations, reported for all nodes and separately for the upper and lower layers.

Configuration	Node group	n	Count ≥10 cm[1]	Count ≥20 cm[1]	Min (cm)	Max (cm)	Mean (cm)	Median (cm)	SD (cm)	Mean absolute (cm)	CV (%)[2]
**V1**	All nodes	232	99	16	−30.70	0	−8.57	−9.40	7,46	8,57	—
V1	Upper layer	208	99	16	−30.70	0	−9.29	−9.75	7,47	9,29	—
V1	Lower layer	24	0	0	−7.10	0	−2.35	0	3,33	2,35	141,4
**V2**	All nodes	287	111	38	−24.70	0	−8.79	−6.70	7,85	8,79	—
V2	Upper layer	268	108	38	−24.70	0	−9.12	−6.85	7,83	9,12	—
V2	Lower layer	19	3	0	−18.80	0	−4.16	0	6,59	4,16	158,4
**V3**	All nodes	290	84	0	−18.60	0	−6.30	−5.20	5,93	6,3	—
V3	Upper layer	266	83	0	−18.60	0	−6.65	−5.75	5,97	6,65	—
V3	Lower layer	24	1	0	−10.70	0	−2.31	0	3,65	2,31	158
**Ammann A4**	All nodes	263	58	27	−32.20	1,8	−6.21	−2.40	8,42	6,28	—
Ammann A4	Upper layer	231	56	26	−32.20	1,8	−6.76	−3.10	8,59	6,83	—
Ammann A4	Lower layer	32	2	1	−28.50	1,6	−2.21	0	5,68	2,31	244,4
**Unibat**	All nodes	113	0	0	−3.20	0	−1.18	−1.10	1,03	1,18	—
Unibat	Upper layer	81	0	0	−3.20	0	−1.09	−1.10	1,06	1,09	—
Unibat	Lower layer	32	0	0	−3.10	−0.20	−1.41	−1.35	0,92	1,41	65,2

Notes: Each node contributes one displacement value. Zero values are retained, and all nodes have equal weight. Minimum, maximum, mean, median and population standard deviation (SD) are calculated from signed displacements. Mean absolute displacement is calculated from their absolute values.

1. Counts refer to absolute displacements, $|U_z| \geq 10$ cm and $|U_z| \geq 20$ cm. These counts are cumulative. The thresholds are descriptive and do not represent serviceability limits.
2. The coefficient of variation is reported only for the lower layer, consistently with the previous tables, and is calculated as $CV = 100\sigma(|U_z|)/\overline{(|U_z|)}$, using the population standard deviation of absolute displacements.
